# Endometrial senescence is mediated by interleukin 17 receptor B signaling

**DOI:** 10.1186/s12964-024-01740-5

**Published:** 2024-07-15

**Authors:** Keiko Kawamura, Yumiko Matsumura, Teruhiko Kawamura, Hiromitsu Araki, Norio Hamada, Kazutaka Kuramoto, Hiroshi Yagi, Ichiro Onoyama, Kazuo Asanoma, Kiyoko Kato

**Affiliations:** 1 Department of Obstetrics and Gynecology, Graduate School of Medical Sciences, Kyushu University, Maidashi 3-1-1, Higashi-ku, Fukuoka, 812-8582 Japan; 2 Department of Business and Technology Management, Faculty of Economics, Kyushu University, Fukuoka, Japan

**Keywords:** IL17RB, IL17B, Endometrial senescence, Aging, NF-κB, SASP, IL1β, Organoid, Macrophage, JNK

## Abstract

**Background:**

We previously identified Il17RB, a member of the IL17 superfamily, as a candidate marker gene for endometrial aging. While IL17RB has been linked to inflammation and malignancies in several organ systems, its function in the endometrium has not been investigated and is thus poorly understood. In the present study, we performed a functional analysis of this receptor with the aim of determining the effects of its age-associated overexpression on the uterine environment.

**Methods:**

We analyzed IL17RB-related signaling pathways and downstream gene expression in an immortalized human endometrial glandular epithelial cell line (“hEM”) forced to express the receptor via lentiviral transduction (“IL17RB-hEM”). We also prepared endometrial organoids from human endometrial tissue sourced from hysterectomy patients (“patient-derived EOs”) and exposed them to cytokines that are upregulated by IL17RB expression to investigate changes in organoid-forming capacity and senescence markers. We analyzed RNA-seq data (GEO accession number GSE132886) from our previous study to identify the signaling pathways associated with altered IL17RB expression. We also analyzed the effects of the JNK pathway on organoid-forming capacity.

**Results:**

Stimulation with interleukin 17B enhanced the NF-κB pathway in IL17RB-hEM, resulting in significantly elevated expression of the genes encoding the senescence associated secretory phenotype (SASP) factors IL6, IL8, and IL1β. Of these cytokines, IL1β inhibited endometrial organoid growth. Bioinformatics analysis showed that the JNK signaling pathway was associated with age-related variation in IL17RB expression. When IL17RB-positive cells were cultured in the presence of IL17B, their organoid-forming capacity was slightly but non-significantly lower than in unexposed IL17RB-positive cells, but when IL17B was paired with a JNK inhibitor (SP600125), it was restored to control levels. Further, IL1β exposure significantly reduced organoid-forming capacity and increased p21 expression in endometrial organoids relative to non-exposure (control), but when IL1β was paired with SP600125, both indicators were restored to levels comparable to the control condition.

**Conclusions:**

We have revealed an association between IL17RB, whose expression increases in the endometrial glandular epithelium with advancing age, and cellular senescence. Using human endometrial organoids as in vitro model, we found that IL1β inhibits cell proliferation and leads to endometrial senescence via the JNK pathway.

**Supplementary Information:**

The online version contains supplementary material available at 10.1186/s12964-024-01740-5.

## Introduction

It is well known that female fertility declines with age [1]. Japanese infertility patients are increasingly turning to assisted reproductive technology (ART) procedures, but pregnancy rates are typically low and miscarriage rates high among women aged 40 and over [2]. Improving treatment outcomes in this demographic remains a major challenge in reproductive medicine. Oocyte senescence is considered the biggest contributor to age-related subfertility: rates of aneuploidy in embryos fertilized in vitro increase with advancing maternal age [3]. Preimplantation genetic testing for aneuploidy (PGT-A) is used in various Western and other countries to avoid the transfer of aneuploid embryos. One study of its clinical efficacy in patients aged 38–41 years observed a significant improvement in delivery rate after the first transfer attempt compared with unscreened patients (52.9% vs. 24.2%); however, the cumulative delivery rate per patient over the following six months was no different between the groups (37.0% vs. 33.3%) [4]. These results show that establishing euploidy via PGT-A does not guarantee a successful pregnancy; in such cases, infertility may be caused by the endometrial environment, i.e., the blastocyst implantation site. Our research group previously analyzed cellular senescence markers in human endometrial stromal cells (ESCs) isolated from tissue biopsied from infertility patients during embryo transfer, including senescence-associated beta-galactosidase (SA-β-gal) and senescence-associated secretory phenotype (SASP) factors, and found evidence of significant elevation in such markers in women who did not become pregnant after embryo transfer compared with those who did [5]. Senescent cells can impair the normal functioning of decidualized ESCs in their vicinity, which was proposed as a cause of repeated implantation failures and recurrent miscarriage in a major review [6]. These findings hint at a link between infertility and endometrial cellular senescence.

It remains inconclusive as to whether biological aging causes functional aging of the uterus. There is some evidence to support the hypothesis that the uterus does not functionally age. In 2014, for example, a patient with Rokitansky syndrome, who congenitally lacked a uterus due to Müllerian duct aplasia, conceived and delivered a child after receiving a uterus donated by a living menopausal woman, in the world’s first live birth resulting from uterus transplantation [7]. Nevertheless, several studies support the opposite conclusion: for example, one study reported higher rates of SA-β-gal positivity in uterine tissue derived from women over 45 years of age [8], while another reported that developmental defects common in embryos from 42 to 54 week-old mice were rescued when they were transferred to 8–12 week-old mice [9].

Previously, our research group attempted to identify genes whose expression patterns in the uterus differed between young mice (5 and 8 weeks old) and aged mice (60–79 weeks old). Not only were mRNA expression levels of three such genes significantly higher in the aged mice relative to the younger ones—IL17RB, CXCL12, and CXCL14—but similar differences in their respective protein expression levels were found in human endometrial lining between patients in their forties and patients in their twenties [10]. This study focuses on the first of these three endometrial aging markers. Interleukin 17 receptor b (IL17RB) belongs to the IL17 superfamily, which consists of six ligands (IL17A–IL17F) and their corresponding receptors (IL17RA–IL17RE) [11]; [12]; [13–23]. It specifically binds to IL17B (encoded at 5q32–34 in humans [14]) and thereby activates downstream signaling [11]. To date, IL17B/IL17RB signaling has been linked to tumor growth and metastasis promotion in various malignancies, including breast [24, 25], pancreatic [26, 27], and lung cancer [28], as well as to inflammation in arthritis and autoimmune diseases [11, 29]. In the present study, we performed a functional analysis of this receptor with the aim of determining the effects of its age-associated overexpression on the uterine environment.

## Materials and methods

### Cell culture

We cultured immortalized human endometrial glandular cells obtained from Kanazawa University’s Department of Obstetrics and Gynecology according to a previously described method [30]. We refer to this cell line as “hEM” below. The cells were cultured at 37 °C under 5% CO_2_ in DMEM/Ham’s-F12 (1:1) medium containing ITS (#I3146, Sigma–Aldrich, St. Louis, MO, USA), 10% fetal bovine serum (FBS), penicillin (100 U/ml), and streptomycin (100 µg/ml) (#15140-122, Gibco/Thermo Fisher Scientific, Waltham, MA, USA).

### Lentiviral production

We used Precision LentiORF IL17RB w/ Stop Codon (#OHS5897-202618086, Horizon Discovery, Waterbeach, UK) as the lentiviral expression vector, with a IL17RB insert. We used a Precision LentiORF RFP positive control (#OHS5832, Horizon Discovery) as the control vector. The lentiviral vectors were packaged and then transduced into HEK293T cells according to a previously described method [31] using Lipofectamine 2000 (#11,668,019, Thermo Fisher Scientific). Forty-eight hours after initiation of the transduction, we collected the culture supernatant and passed it through a 0.45 μm filter, and then added the lentivirus-containing filtrate to hEM cultures in the presence of polybrene, to a final concentration of 8 ng/µl.

### Protein extraction and western blotting

We washed the cells in phosphate-buffered saline (PBS) cooled them to 4 °C, then lysed them in lysis buffer (#C2978, Sigma–Aldrich) containing a protease inhibitor cocktail (#P8340, Sigma–Aldrich) and phosphatase inhibitor cocktail (#07574-61, Nacalai Tesque, Kyoto, Japan). Electrophoresis was run in 10% precast gradient polyacrylamide gels (5 µg protein per lane). We transferred the separated proteins from the gels onto nitrocellulose membranes at 20 V overnight, after which we blocked the membranes either for 20 min with 5% milk or for 1 h with polyvinylidene fluoride (PVDF) blocking reagent (#NYPBR01, Toyobo, Osaka, Japan). We added the following primary antibodies and allowed the samples to react overnight at 4 °C: IL17RB (1:1000, #GTX127368, GeneTex, Irvine, CA, USA), Phospho-IκBα (Ser32) (14D4) mAb (1:1000, #2859, Cell Signaling Technology, Danvers, MA, USA), IκBα (44D4) mAb (1:1000, #4812, Cell Signaling Technology), Phospho-SAPK/JNK (Thr183/Try185) mAb (1:2000, #9255, Cell Signaling Technology), SAPK/JNK mAb (1:1000, #9252, Cell Signaling Technology), GAPDH (1:5000, #015-25473, Fujifilm Wako Pure Chemical Corporation, Osaka, Japan). Next, we washed the membranes with TBST (a mixture of tris-buffered saline and polysorbate 20) and then reacted them with one of the following secondary antibodies for 1 h at room temperature: Anti-Rabbit or Anti-Mouse IgG (whole molecule)-peroxidase antibody produced in goat (1:5000, #A6154 or #A4416, Sigma–Aldrich). We detected specific protein bands using SuperSignal West Dura Chemiluminescent Substrate (#34,080, Thermo Fisher Scientific).

### NF-κB reporter assay

We simultaneously transfected the cells with pNL3.2 NF-κB-RE vector (#N1111, Promega, Madison, WI, USA) and pGL4.53 (Luc2/PGK) vector (#E5011, Promega) using FuGENE HD Transfection Reagent (#E2313, Promega). We seeded them in 96-well plates 24 h after transfection and, after another 24 h, treated them with IL17B at one of the following concentrations: 0, 20, or 200 ng/ml. We evaluated luciferase activity 6 h after IL17B treatment using the Nano-Glo Dual-Luciferase Reporter Assay System (#N1610, Promega) and a VICTOR Nivo Multimode Microplate Reader (PerkinElmer, Waltham, MA, USA).

### RNA purification and RT-qPCR

We purified RNA samples using a RNeasy Plus Mini Kit (QIAGEN, North Rhine-Westphalia, Germany) and subjected them to a one- or two-step quantitative reverse transcription–polymerase chain reaction (RT-qPCR). For the two-step RT-qPCR, we performed the reverse transcription using ReverTra Ace qPCR RT Master Mix (#FSQ-201, Toyobo) and real-time PCR using specific primers for each gene (Table 1). We ran the experiments using TB Green premix Ex Taq TM II (#RR820A, Takara, Shiga, Japan) or a SYBR Green RT-PCR kit (#204,243, QIAGEN) on the CFX Connect Real-Time PCR System (BioRad, Hercules, CA, USA) operated according to the manufacturer’s protocol. We quantified the expression levels of each target gene relative to those of endogenous controls (*HPRT-1*, 18SrRNA) using the ∆∆CT method (control expression: 1.0).

**Table 1:** Primers used for RT–PCR

Gene	Primer		Reference
	Forward (3′–5′)	Reverse (5′–3′)	
IL17RB	AGGGACCTCCGAGTAGAACC	CTTGGTGGCCTTCAACAAGC	[32]
IL6	AATTCGGTACATCCTCGACGG	GGTTGTTTTCTGCCAGTGCCT	[33]
CXCL8 (IL8)	AAGGAAAACTGGGTGCAGAG	ATTGCATCTGGCAACCCTAC	[34]
IL1β	AAGCTGATGGCCCTAAACAG	AGGTGCATCGTGCACATAAG	[35]
CDKN1A (p21)	TGGACCTGTCACTGTCTTGT	TCCTGTGGGCGGATTA	[36]
IL17B	CCCAGAGAAAGTGTGAGGTCAA	GGTCCTCCTGCATGGTGAAG	[26]
HPRT-1	GGCAGTATAATCCAAAGATGGTCAA	GTCAAGAGCATATCCTACAACAAAC	-
18SrRNA	GTAACCCGTTGAACCCCATT	CCATCCAATCGGTAGTAGCG	[37]

### Three-dimensional organoid cultures derived from human endometrial tissue

We prepared three-dimensional (3D) organoid cultures from human endometrial tissue donated by hysterectomy patients. This model is hereafter referred to as “patient-derived endometrial organoids (EOs)”. All donors gave their written informed consent, and their background information is shown in Table 2. These experiments were approved by the institutional review board of Kyushu University (approval no. 22087-00). To examine the in vivo morphology of the tissue, we fixed part of each specimen in formalin, embedded it in paraffin, sliced it into 4 μm sections, and stained these with hematoxylin–eosin (HE) for histological observation via optical microscopy. We collected the rest of the tissue in 4 °C PBS, centrifuged it at 2000 × *g* for 6 min to remove the supernatant, and then treated it with lysis buffer to remove erythrocytes. We prepared an enzymatic mixture by adding DNase (#2270A, Takara) and 10 µM Y-27,632 dihydrochloride (#034-24024, Fujifilm Wako Pure Chemical Corporation) to 5 mg/ml collagenase type II (#LS004197, Worthington Biochemical Corporation, Lakewood, NJ, USA) in HBSS (#17461-05, Nacalai Tesque). We treated the pellet from the previous step with this enzymatic mixture at 37 °C for 1–1.5 h, agitating it by pipette every 15 min. Next, we used 100 μm filters (#93,100, SPL Life Sciences, Gyeonggi-do, Korea) and TrypLE Express (#12604-021, Gibco) to disaggregate the tissue into individual cells and collect epithelial cells.

**Table 2:** Background of hysterectomy patients

ID	Age	BMI	Indication/diagnosis	Reproductive history	Menstrual stage	Assay
EM_1	52	17.1	Leiomyoma	G0P0	Proliferative	HE stain
EM_2	50	38.1	Leiomyoma	G0P0	Proliferative	Formation, qPCR, β–gal
EM_3	43	20	Leiomyoma	G1P0	Proliferative	Formation, qPCR, β–gal
EM_4	33	26.9	Leiomyoma	G4P2	Proliferative	β–gal
EM_5	43	18.3	CC stage IA1	G4P2	Secretory	Formation, qPCR
EM_6	32	17.9	CC stageIB3	G0P0	Proliferative	β–gal、apoptosis
EM_7	41	17.5	CC stage IA1	G4P3	Secretory	IF
EM_8	44	21.1	CC stage IB1	G3P3	Secretory	IF
EM_9	33	29.2	CC stage IB2	G0P0	Proliferative	Formation, time–lapse
EM_10	40	25	CC stage IB3	G0P0	Secretory	Formation, qPCR

*G* gravidity, *P* parity, *CC* Cervical Cancer

We pipetted the isolated cells onto a 24-well plate at a seed density of 3.5 × 10^4^ cells per 10 µL Matrigel (#356,231, Corning, Corning, NY, USA). We then cultured the cells in organoid expansion medium (“ExM”) prepared according to a previously described method [38] with replacement every 2–3 d (500 µl/well; composition shown in Table 3).

**Table 3:** Compotision of basal medium and expansion medium

Basal medium (DMEM/F12)			
Product	Company	Product number	Final concentration
DMEM/Ham's F–12	Gibco	12634–010	
Pencillin–streptomycin	Gibco	15140–122	
HEPES	Nacalai Tesque	17557–94	10 mM
Glutamax	Gibco	35050–06	2 mM
Expansion medium (ExM)			
Product	Company	Product number	Final concentration
Basal medium			46.60%
B27 Supplement (50×)	Gibco	2389219	2%
N–acetylcysteine	Wako	015–05132	1 mM
Recombinant Human FGF10	Peprotech	100–26	10 ng/ml
Nicotinamide	Wako	141–01202	10 mM
A83–01	Nacalai Tesque	19692–54	500 nM
SB202190	Sigma	S7067	10 µM
Y–27632	Wako	034–24024	10 µM
Recombinant EGF	Peprotech	100–15	50 ng/ml
Primocin	InvivoGen	ant–pm	50 µg/ml
Afamin Wnt3A*			50%

*We used L-WRN cell culture supernatant containing Wnt3a, R-spondin, and Noggin [39][40]

We examined organoid morphology via HE staining. We stripped each culture of Matrigel by adding Cell Recovery Solution (#354,253, Corning), pipette-agitated it, washed it with basal medium, and then centrifuged them to prepare a pellet. The result was jellified using iPGell (Nippon Genetics Corporation, Tokyo, Japan), according to the manufacturer’s protocol, followed by fixation in 4% paraformaldehyde. The solidified suspension was then embedded in paraffin, sliced into 4 μm sections, HE-stained, and observed under an optical microscope.

### Organoid-forming efficiency analysis

We treated the cultured organoids with Cell Recovery Solution to remove the Matrigel, agitated them by pipetting, and trypsinized them using TrypLE Express. We counted the disaggregated cells using a hematocytometer grid and then seeded them on a 24-well plate at a density of 3.5 × 10^4^ cells per 10 µl Matrigel. To each well, we added 500 µl ExM containing one of three recombinant human cytokines: IL6 (#200-06, PeproTech, Cranbury, NJ, USA), IL8 (#200-08, PeproTech), or IL1β (#200-01B, PeproTech). We maintained the cultures for 10–20 d, exchanging the cytokine-containing medium every 48 h. Finally, we observed them using an all-in-one fluorescence microscope (BZ-X710: Keyence, Osaka, Japan) to check for cytokine-associated differences in organoid-forming efficiency, which we quantified as the number of organoids (i.e., ≥ 20 μm in diameter) per unit volume in Z-stacked images (2.28 × 10^−1^mm^3^) using BZ-X analysis software (BZ-X700 Analyzer, Keyence).

### SA-β-gal assay

We measured senescence-associated β-galactosidase (“SA-β-gal”) using a SPiDER-β-Gal detection kit (#SG-03, Dojindo, Kumamoto, Japan) according to the manufacturer’s protocol. Organoids seeded and cultured in a 48-well plate were immuno-labeled after ExM removal and washing. We added Cell Recovery Solution to remove the Matrigel, and then dispersed the organoids via pipette agitation and trypsinized them using TrypLE Express. We washed the samples and passed them through a 40 μm filter; dead cells were removed using propidium iodide (#P-4170, Sigma–Aldrich). We measured SPiDER-β-Gal fluorescence using a BD FACSMelody Cell Sorter (BD Biosciences, Franklin Lakes, NJ, USA).

### SA-β-gal staining

We seeded mock-hEM and IL17RB-hEM in a 6-well plate (5.0 × 10^4^ cells/well). IL17B (100ng/ml) was added to the culture medium and the cells were passaged repeatedly. After reaching the target number of passages, cells were stained according to the protocol of the Cellular Senescence Assay Kit (#CBA-230, CELL BIOLABS, INC., San Diego, CA, USA). Senescent cells were identified based on blue cytoplasmic staining under bright field microscopy (BZ-X700, Keyence): percentages of senescent cells per unit area (number of senescent cells/total cell count) was measured and compared with control conditions.

### Apoptosis assay

Following organoid seeding and culture in a 48-well plate, we removed the ExM and washed the organoids. We added Cell Recovery Solution to remove the Matrigel from the plate, and then dispersed the organoids via pipette agitation and trypsinized them using TrypLE Express. The cell suspension was stained according to the protocol of the MEBCYTO Apoptosis Kit (#4700, MBL, Tokyo, Japan) and analyzed using a BD FACSMelody Cell Sorter.

### Immunocytochemistry

We seeded the cells in collagen-coated eight-well chamber slides (3.0 × 10^4^ cells/well), incubated them for 24 h at 37 °C under 5% CO_2_, and then incubated them for another 24 h in the presence or absence of IL17B (100 ng/ml). We fixed the cultures in 4% paraformaldehyde (#09154-85, Nacalai Tesque)/PBS, permeabilized them with 0.25% Triton X/PBS for 15 min at room temperature, and then blocked them with Protein Block Serum-Free (#X0909, Dako North America, Carpinteria, CA, USA) for 10 min at room temperature, with each step preceded by washing the sample three times with PBS. Next, we reacted the cells with primary antibody overnight at 4°C (anti-NF-κB p65, 1:100, #sc-372, Santa Cruz Biotechnology, Dallas, TX, USA), tripled-washed them in PBS, reacted them with secondary antibody for 30 min at room temperature out of direct light (Alexa Fluor 488 donkey anti-rabbit IgG(H + L), 1:500, #A21206, Invitrogen/Thermo Fisher Scientific), and triple-washed them in PBS again. We treated the reacted cells with ProLong Gold antifade reagent with DAPI (#P36935, Invitrogen/Thermo Fisher Scientific), covered them with a glass coverslip, and imaged the resulting fluorescence using a C2 confocal microscope (Nikon, Tokyo, Japan). We evaluated the intensity of the fluorescence in the images thus obtained using ImageJ software [41].

### Immunofluorescence

We analyzed specimens of normal human endometrial tissue using immunofluorescence. These experiments were approved by the institutional review board of Kyushu University (approval no. 622-00). First, we prepared 4 μm paraffin sections, allowed them to air dry, and then deparaffinized them. We retrieved the antigens via microwave heating in 0.1% sodium azide at pH 9.0. We allowed them to cool for 20 min at room temperature and then blocked them for 10 min using Protein Block Serum-Free (#X0909, Dako North America). The samples were then allowed to react overnight at 4 °C with the following primary antibodies: CD68 monoclonal antibody (1:100, #76,437, Cell Signaling Technology), IL1β Mouse mAb (1:100, #12242S, Cell Signaling Technology), and anti-IL17B (1:100, #NBP2-11672, Novus Biologicals, Englewood, CO, USA). After washing them with PBS, we incubated them for 30 min at room temperature with the following secondary antibodies: Alexa Fluor 488 donkey anti-rabbit IgG(H + L) and Alexa Fluor 647 goat anti-mouse IgG(H + L) (1:1000 each, #A21206 and #A21236, Invitrogen/Thermo Fisher Scientific). We washed the reacted tissue with PBS, treated it with ProLong Gold antifade reagent with DAPI (#P36935, Invitrogen/Thermo Fisher Scientific), covered it with a glass coverslip, and imaged the fluorescence using a C2 confocal microscope (Nikon).

### Monocyte separation

We used peripheral blood collected from laboratory volunteers using a Lymphoprep Tube (CosmoBio, Carlsbad, CA, USA) as a source of mononuclear cells. We isolated classic monocytes (CD14^+^/CD16^−^) from samples using the EasySep Human Monocyte Isolation kit according to the manufacturer’s protocol (#ST-19,359, Stemcell Technologies, Vancouver, BC, Canada). Once isolated, we cultured these cells in RPMI 1640 (#30264-56, Nacalai Tesque) supplemented with 50 ng/ml M-CSF (#300 − 25, PeproTech) to induce them to differentiate into macrophages. On day 6 of the culture, we activated the macrophages by adding lipopolysaccharide (LPS) and Pam3CSK4 (both 1 µg/ml; #L2630 and #P1585, Sigma–Aldrich). On day 7, we collected them for RNA extraction and, to confirm successful differentiation into macrophages, we trypsinized some cells and collected them from the dish, washed them, suspended them in FACS Buffer (PBS containing 2% FBS; 1.0 × 10^6^ cells/sample), and blocked them for 15 min using FcR Blocking Reagent (#130-059-901, Miltenyi Biotec, North Rhine-Westphalia, Germany). We then washed them, suspended them in 100 µl FACS Buffer, and stained them for 15 min with CD68 monoclonal antibody (#76,437, Cell Signaling Technology) and FITC anti-human CD80 (#305,206, BioLegend, San Diego, CA, USA). Next, we washed them with FACS Buffer and then stained them for 15 min with PE Donkey anti-rabbit IgG (#406,421, BioLegend). Finally, we analyzed the cells using a BD FACSMelody Cell Sorter.

### Time-lapse photography

We disaggregated endometrial tissue specimens donated by hysterectomy patients using the method described above. Each sample (1.0 × 10^7^ cells) was washed, suspended in FACS Buffer, and blocked with anti-FcR mAb for 15 min. Next, we suspended it in 100 µl FACS Buffer and stained it for 15 min with FITC anti-human CD9 (#312,104 BioLegend), PE/Cyanine7 anti-human CD10 (#312,214, BioLegend), and APC anti-human IL17RB (#FAB 1207 A, R&D Systems, Minneapolis, MN, USA), after which we removed dead cells using propidium iodide. We then sorted the cells into IL17RB-positive and IL17RB-negative groups using the BD FACSMelody Cell Sorter. We seeded the two groups separately in 48-well plates at a density of 4.5 × 10^3^ cells per 5 µl Matrigel and cultured them in ExM (250 µl/well) containing either IL17B only (100 ng/ml), SP600125 only (5 µM), or both. We grew these cultures at 37 °C under 5% CO_2_ for 7 d, during which we recorded them using time-lapse photography under a fluorescence microscope (BZ-X800, Keyence). We quantified the cultures in terms of organoid count (i.e., organoids ≥ 20 μm in diameter) per unit volume in Z-stacked images using a BZ-X700 microscope and BZ-X analysis software (BZ-X700 Analyzer, Keyence).

### Statistical analysis

We performed all statistical analyses under advice from a professional clinical statistician, using GraphPad Prism 7 software on Windows 7.02 (GraphPad Software, San Diego, CA, USA). We used Student’s *t*-tests for comparisons between two independent groups and Dunnett’s multiple comparison test for multiple comparisons. Results data are presented with standard deviations; *P* < 0.05 was considered statistically significant. All analyses included post hoc tests.

## Results

### IL17B/IL17RB signaling pathway analysis in IL17RB-hEM

**Fig. 1:**
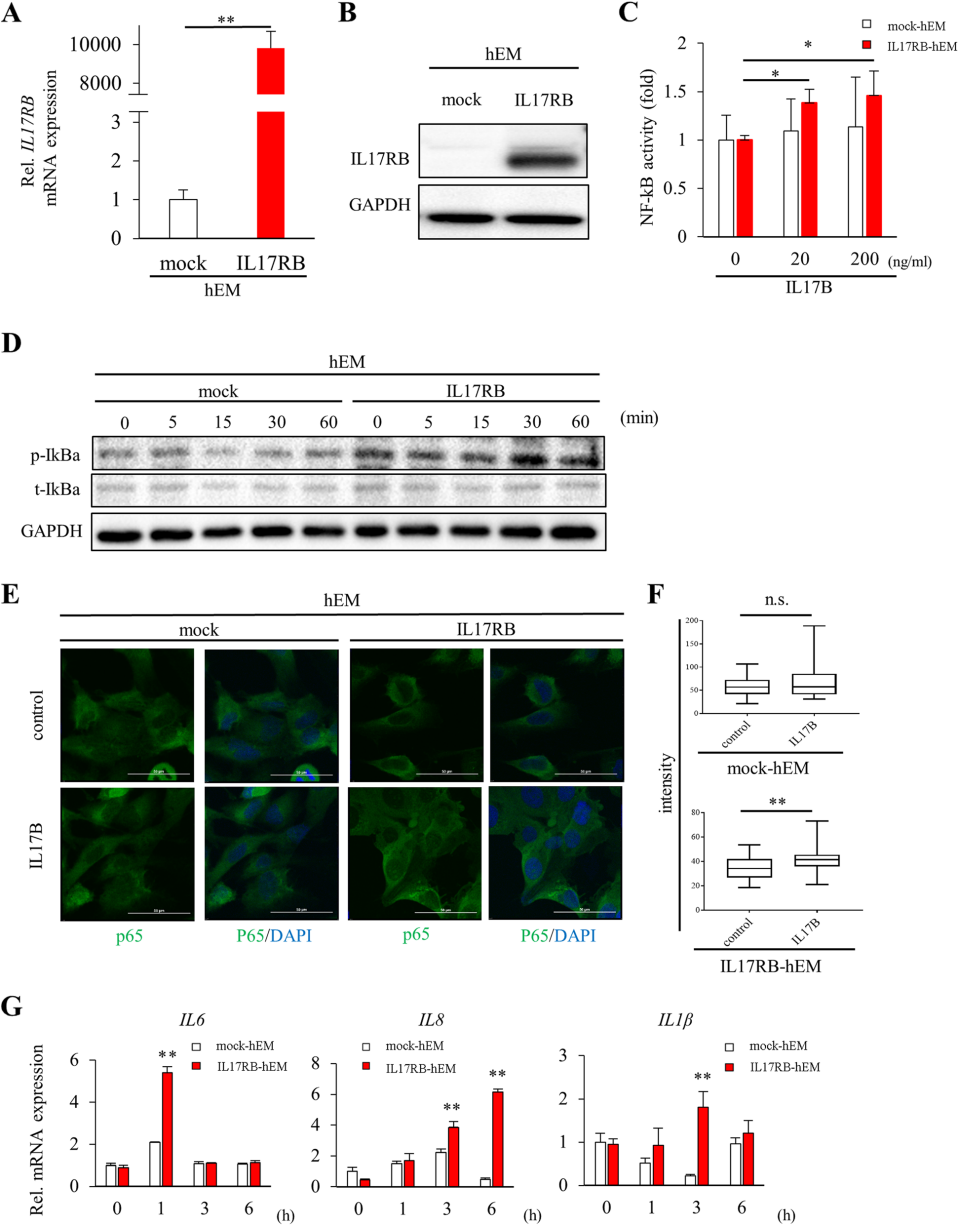
IL17B/IL17RB signaling pathway analysis (hEM). Experiments compared immortalized human endometrial glandular cells forced to express IL17RB via a lentiviral vector (“IL17RB”) with mock-transfected hEM (“mock”). (A) *IL17RB* mRNA expression as quantified via RT-qPCR relative to an endogenous control (*HPRT1*). Error bars denote standard deviation (*n* = 3). **P < 0.01. (B) IL17RB protein expression as evaluated via Western blotting. GAPDH was used as the loading control. (C) NF-κB activity 6 h after IL17B stimulation as measured via dual luciferase assay. Error bars denote standard deviation (*n* = 3). *P < 0.05. (D) IκBα phosphorylation after IL17B stimulation (100 ng/ml) as evaluated via Western blotting. p-IκBα: phosphorylated IκBα, t-IκBα: total IκBα, GAPDH: loading control. (E) Immunofluorescent staining of p65 nuclear translocation 16 h after IL17B stimulation (100 ng/ml). Scale bars: 50 μm. (F) Nuclear intensity in p65-immunostained images as measured using ImageJ. Box plots representing the mean intensity per pixel within the nucleus of a single cell are shown. ** *P* < 0.01, n.s., not significant. (G) *IL6*, *IL8*, and *IL1β* mRNA expression. Expression was measured via RT-qPCR at 0 (baseline), 1, 3, and 6 h after IL17B stimulation (100 ng/ml). Endogenous control: *HPRT1*. Error bars denote standard deviation (*n* = 3). **P < 0.01

We analyzed the function of IL17RB in the endometrium using a series of forced-expression experiments. Specifically, we forced immortalized hEM to express the receptor via transduction using a lentiviral vector with a IL17RB insert (“IL17RB-hEM”) and compared their expression patterns with those of mock-transfected hEM. We confirmed successful transduction based on substantially higher levels of *Il17RB* mRNA transcript and IL17RB protein product (Fig. 1A–B). To verify this receptor’s ability to relay intracellular signals, we examined the activation patterns of NF-κB, a transcription factor and important regulator of the inflammatory response that is thought to be downstream of it. We confirmed via dual luciferase assay that NF-κB activation was enhanced by stimulation with the receptor ligand (IL17B) in a statistically significant and concentration-dependent manner (Fig. 1C). In Western blotting, only IL17RB-hEM exhibited time-dependent IKBα phosphorylation following exposure to 100 ng/ml IL17B (Fig. 1D). Next, we verified NF-κB nuclear translocation via p65 immunocytochemistry, i.e., by quantifying the signal intensity of immuno-labeled p65 within cell nuclei after IL17B stimulation (100 ng/ml) and non-stimulation (control) in IL17RB- and mock-hEM using ImageJ software. IL17B exposure significantly enhanced intranuclear fluorescence compared to non-exposure only in IL17RB-hEM (Fig. 1E-F), indicating that ligand-induced activation of the NF-κB pathway is enhanced only in endometrial cells that overexpress IL17RB. In addition to various proinflammatory genes, NF-κB has been widely reported to induce the expression of senescence-associated secretory phenotype (SASP), a collection of factors such as cytokines and chemokines that are secreted by senescent cells in response to genotoxic stress, which has been linked to inflammation and carcinogenesis [42]. We confirmed that IL17B stimulation significantly enhanced the gene expression of three representative SASP factors—IL6, IL8, and IL1β—in IL17RB-hEM at several timepoints after exposure (IL6: 1 h; IL8: 3 h, 6 h; IL1β: 3 h; *P* < 0.01; Fig. 1G), suggesting that NF-κB signaling can be amplified and SASP can be induced via IL17RB overexpression in endometrial cells.

### Endometrial response to cytokines downstream of IL17RB in patient-derived EOs

The details of the patients who supplied the patient-derived EOs we prepared are summarized in Table II. We incubated the disaggregated tissue in ExM and were able to confirm the formation of spherical organoids after 10–14 days of culture (Supplementary Fig. S1A–B). Morphologically, their tubular structure resembled that of the biological endometrial glands visible in HE slides of donor tissue (Supplementary Fig. S1C).

**Fig. 2:**
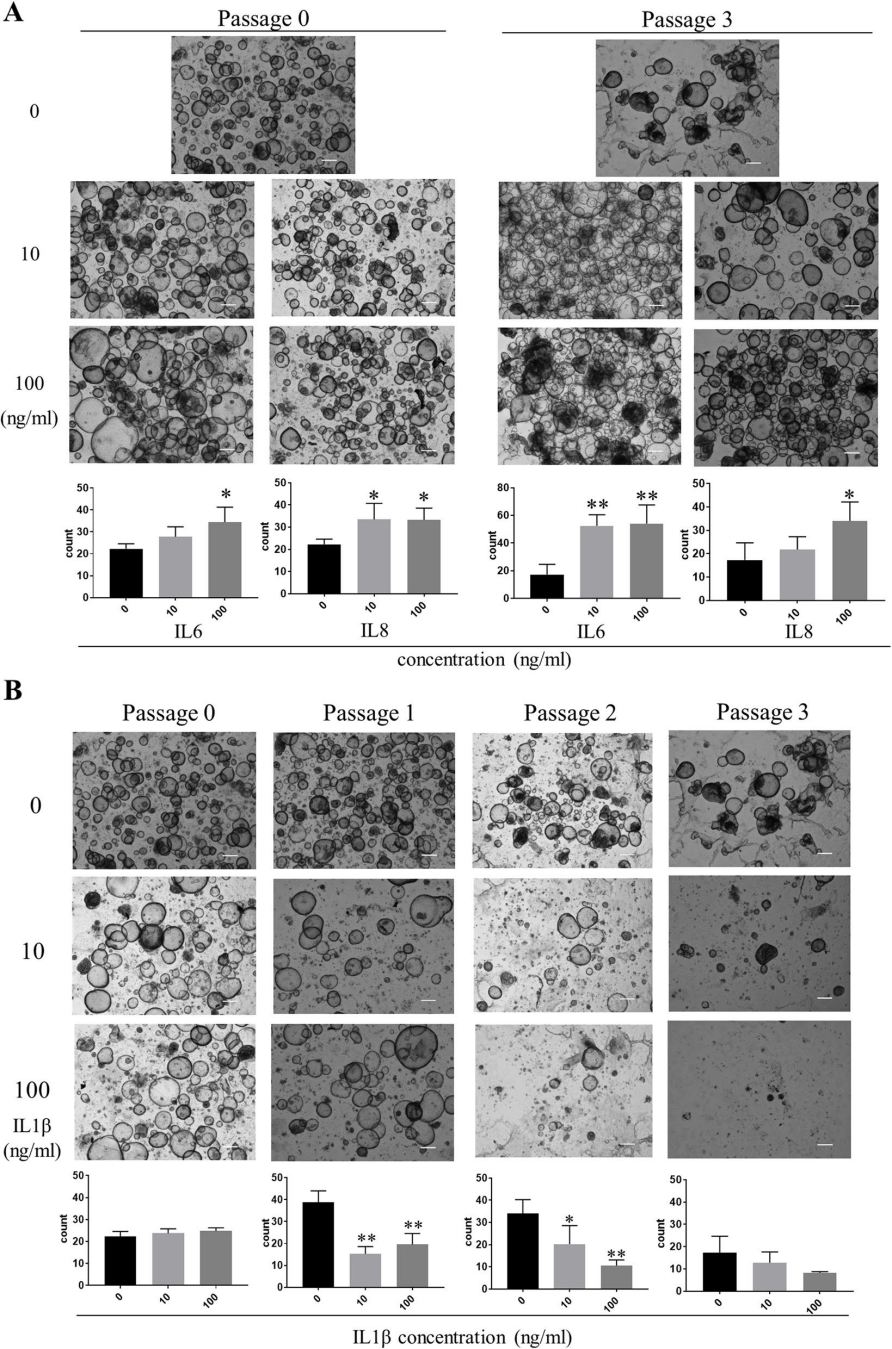
Endometrial response to cytokines downstream of IL17RB (patient-derived EOs). This series of experiments used endometrial organoids grown from tissue specimens from hysterectomy patients. (A) Endometrial organoids (EM_2) cultured in ExM containing IL6 (10, 100 ng/ml) or IL8 (10, 100 ng/ml). Passage 0 and 3 images were taken on day 16 and 17 of culture, respectively. Bar graphs show the numbers of organoids (diameter ≥ 20 μm) counted in each passage and exposure condition (IL6: 0, 10, 100 ng/ml; IL8: 0, 10, 100 ng/ml). Error bars denote standard deviation (*n* = 4 independent locations). Scale bars: 300 μm. (B) Endometrial organoids (EM_2) cultured in ExM containing IL1β (0, 10, 100 ng/ml). Passage 0, 1, 2, and 3 images were taken on day 16, 10, 11, and 17 of culture, respectively. Bar graphs show organoid count (diameter ≥ 20 μm) by passage and exposure condition (IL1β: 0, 10, 100 ng/ml). Error bars denote standard deviation (*n* = 4 independent locations). Scale bars: 300 μm

Next, we examined how the endometrial cells were affected by IL6, IL8, and IL1β—the three cytokines whose expression was enhanced under forced IL17RB expression in the preceding experiments (Fig. 1). We embedded the cells in Matrigel (3.5 × 10^4^ cells/culture) and cultured them in ExM containing 0 ng/ml (control), 10 ng/ml, or 100 ng/ml one of the three cytokines. To confirm the effects of sustained cytokine exposure on organoid-forming capacity, we passaged the cells by disaggregating the resulting spheroids every 10–20 days and embedding the same number of cells in Matrigel, and then compared their organoid-forming capacity across conditions at each passage. When they were cultured in ExM containing IL6, their organoid-forming capacity was significantly enhanced at passage 0 (100 ng/ml vs. control) and even at 10 ng/ml after repeated passaging. With IL8, it was significantly enhanced at passage 0 in both the 10 ng/ml and 100 ng/ml conditions, but by passage 3, this difference persisted only in the 100 ng/ml condition (Fig. 2A). When cultured in ExM containing IL1β, their organoid-forming capacity was unaffected at passage 0, but significantly inhibited at both concentrations in passages 1 and 2. These differences were nonsignificant by passage 3 because the organoid-forming capacity in the control had reduced relative to previous passages (Fig. 2B). The same pattern was corroborated in cultures originating from different donors (Supplementary Fig. S2A–B). These results suggest that long-term IL1β exposure may suppress the organoid-forming capacity of the endometrium.

**Fig. 3:**
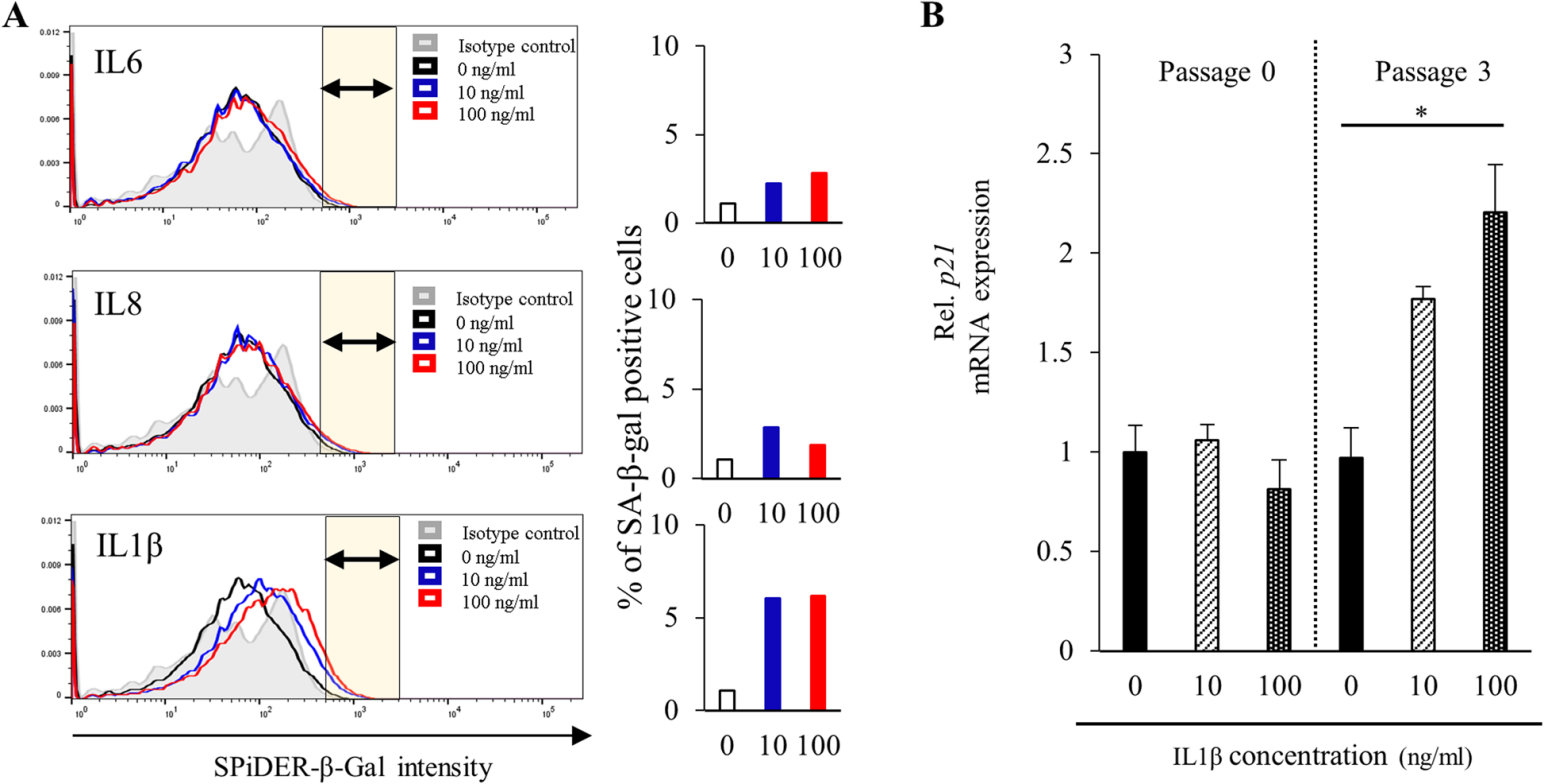
**A** SPiDER-β-Gal flow cytometry of endometrial organoids (EM_2, passage 3). Horizontal axis: SPiDER-β-Gal intensity; vertical axis: unit area. Bar graphs show the percentage of the area under each curve that is brighter than in the negative control (yellow area: SA-β-gal-positive cells). **B** *p21* mRNA expression in patient-derived endometrial organoids (EM_2; passages 0, 3) cultured in ExM containing IL1β (0, 10, 100 ng/ml). Expression was quantified via RT-qPCR relative to an endogenous control (18 S rRNA). Error bars denote standard deviation (*n* = 3). * *P* < 0.05, **P < 0.01

Next, we evaluated the effects of the same cytokines on cellular senescence in patient-derived EOs in terms of the resulting expression levels of SA-β-gal. We subjected the specimens to fluorescence staining using a SPiDER-β-Gal assay kit and evaluated their fluorescence intensity via flow cytometry. Relative to the control condition (non-exposure), SA-β-gal immunofluorescence did not increase in response to either IL6 or IL8 exposure; however, it did increase in response to IL1β. Organoids cultured in its presence had the largest percentages of SA-β-gal–positive cells across conditions (Fig. 3A). We found similar patterns in samples from multiple donors (Supplementary Fig. S3A-B). These results thus suggest that endometrial senescence can be induced by long-term IL1β exposure. These findings prompted us to examine the expression of the senescence marker p21 in endometrial organoids subjected to long-term IL1β exposure. In passage 0, IL1β did not significantly alter p21 expression relative to non-exposure at either concentration; however, by passage 3, p21 levels had become significantly higher in the 100 ng/ml condition (Fig. 3B). Again, we observed similar patterns in samples from multiple donors (Supplementary Fig. S2C–D). To exclude apoptosis as the cause of the reduced organoid-forming capacity induced by long-term exposure to IL1β, we conducted an apoptosis assay. Endometrial organoids exposed to IL1β showed greater proportions of SA-β-gal positive cells compared to the IL6 and IL8 exposure conditions; however, their rates of early and late apoptosis were no higher than in the control condition (Supplementary Fig. S4A–B). Our findings thus support the hypothesis that endometrial senescence can be induced by chronic IL1β stimulation.

### IL17B and IL1β are expressed by activated peripheral-blood–derived macrophages

**Fig. 4:**
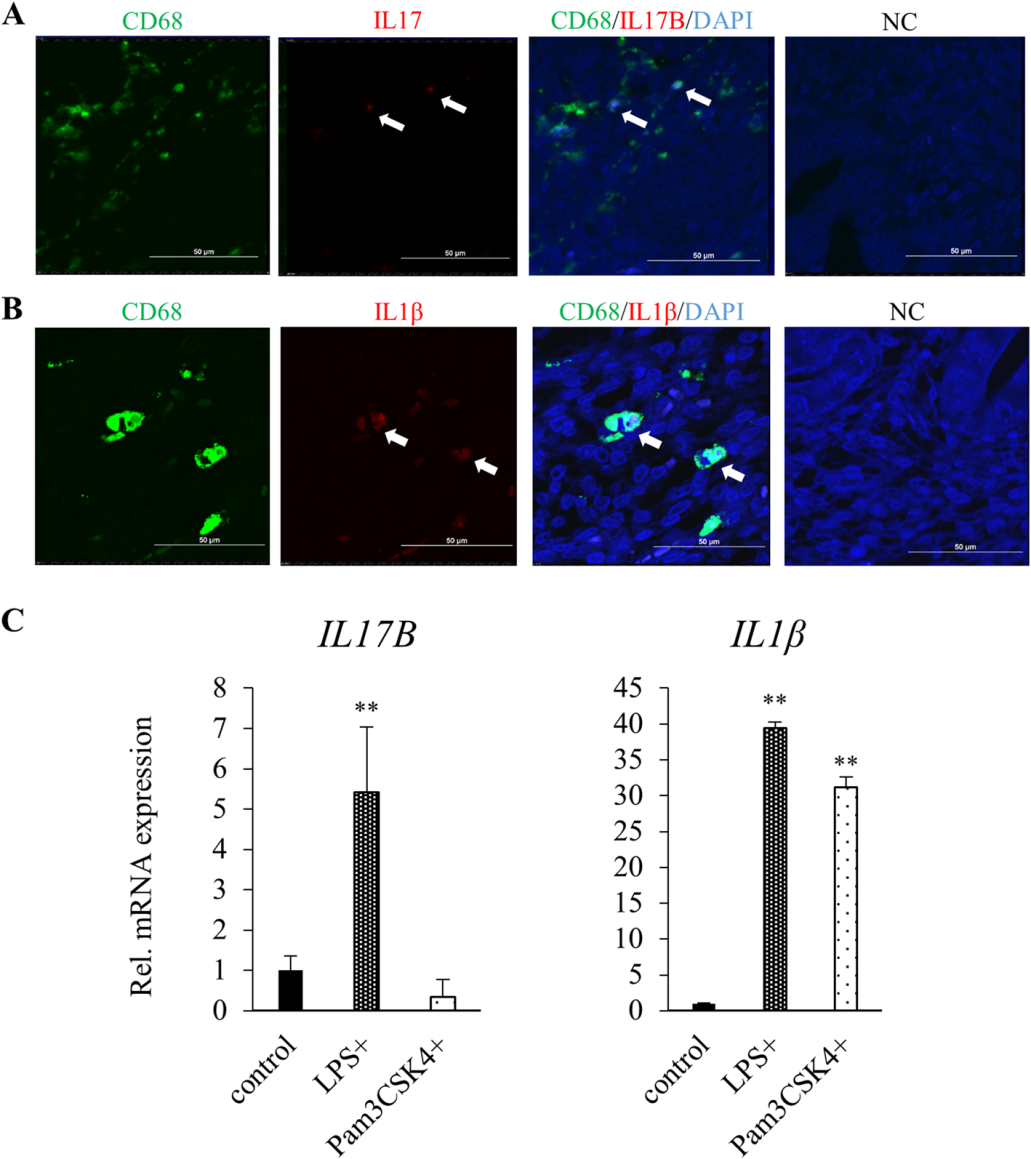
**A** Fluorescent double immunostaining (CD68 + IL17B) of sections of healthy endometrial tissue from a female patient in her forties (EM_7). NC: Negative Control. Scale bars: 50 μm. **B** Fluorescent double immunostaining (CD68 + IL1β) of sections of healthy endometrial tissue from a female patient in her forties (EM_8). NC: Negative Control. Scale bars: 50 μm. (C) *IL17B* and *IL1β* mRNA expression measured via RT-qPCR in macrophages derived from peripheral blood monocytes activated with LPS (1000 ng/ml) and Pam3CSK4 (1000 ng/ml). Endogenous control: *HPRT1*. Error bars denote standard deviation (*n* = 3). ** *P* < 0.01

The next series of experiments focused on identifying the cell population that expresses IL17B, the ligand of IL17RB. From our previous work, we knew that the endometrial tissue of women in their forties tends to contain higher proportions of cells expressing CD68, a macrophage surface marker, than that of women in their twenties. Here, we evaluated the expression patterns of CD68 and two cytokines in histology sections of endometria from women in their forties using two forms of fluorescent double immunostaining: CD68 + IL17B (Fig. 4A) and CD68 + IL1β (Fig. 4B). CD68-positive cells typically co-expressed IL17B and IL1β, suggesting that both of these cytokines are secreted by macrophages in endometrial tissue.

Next, we tested whether these cytokines are secreted by macrophages in general. We induced peripheral-blood monocytes isolated from laboratory volunteers to differentiate into macrophages by culturing them in liquid medium supplemented with M-CSF, with replacement every two days. Cells displaying macrophage-like morphology were visible by day 6 of culture (Fig. S4B), and we activated them by adding a TLR4 agonist and a TLR1/2 agonist to the medium (LPS and Pam3CSK4, respectively; Fig. S4A). On day 7 of culture, we collected the macrophages for RNA extraction and used flow cytometry to verify their expression of CD68 and CD80, an activation marker (Fig. S4C). Cells treated with LPS showed elevated expression of both IL17B and IL1β mRNA relative to unstimulated macrophages, while those treated with Pam3CSK4 exhibited upregulation of only IL1β mRNA (Fig. 4C). Taken together, these findings support the conclusion that macrophages secrete IL17B, the specific ligand of IL17RB, along with IL1β, a potent inducer of endometrial senescence.

### IL17RB signaling induces senescence in endometrial cells

To analyze the relationship between IL17RB signaling and endometrial senescence, we stained mock-hEM and IL17RB-hEM for SA-β-gal after repeated passaging in the presence of IL17B (0 ng/ml, 100 ng/ml). SA-β-gal positive cells, identified by blue cytoplasmic staining, were observed in IL17B-treated IL17RB-hEM after 30 passages, and made up a significantly higher proportion of cells compared to the first passage (Supplementary Fig. S6A, S6B). Next, we analyzed the expression of the senescence marker p21 using RT-qPCR. After 30 passages, only IL17B-treated IL17RB-hEM showed significantly elevated p21 expression with respect to the first passage (Supplementary Fig. S6C). Taken together, these findings suggest that senescence in endometrial cells is induced via IL17RB signaling.

### IL17RB signaling is mediated by the c-Jun N-terminal Kinase (JNK) pathway

**Fig. 5:**
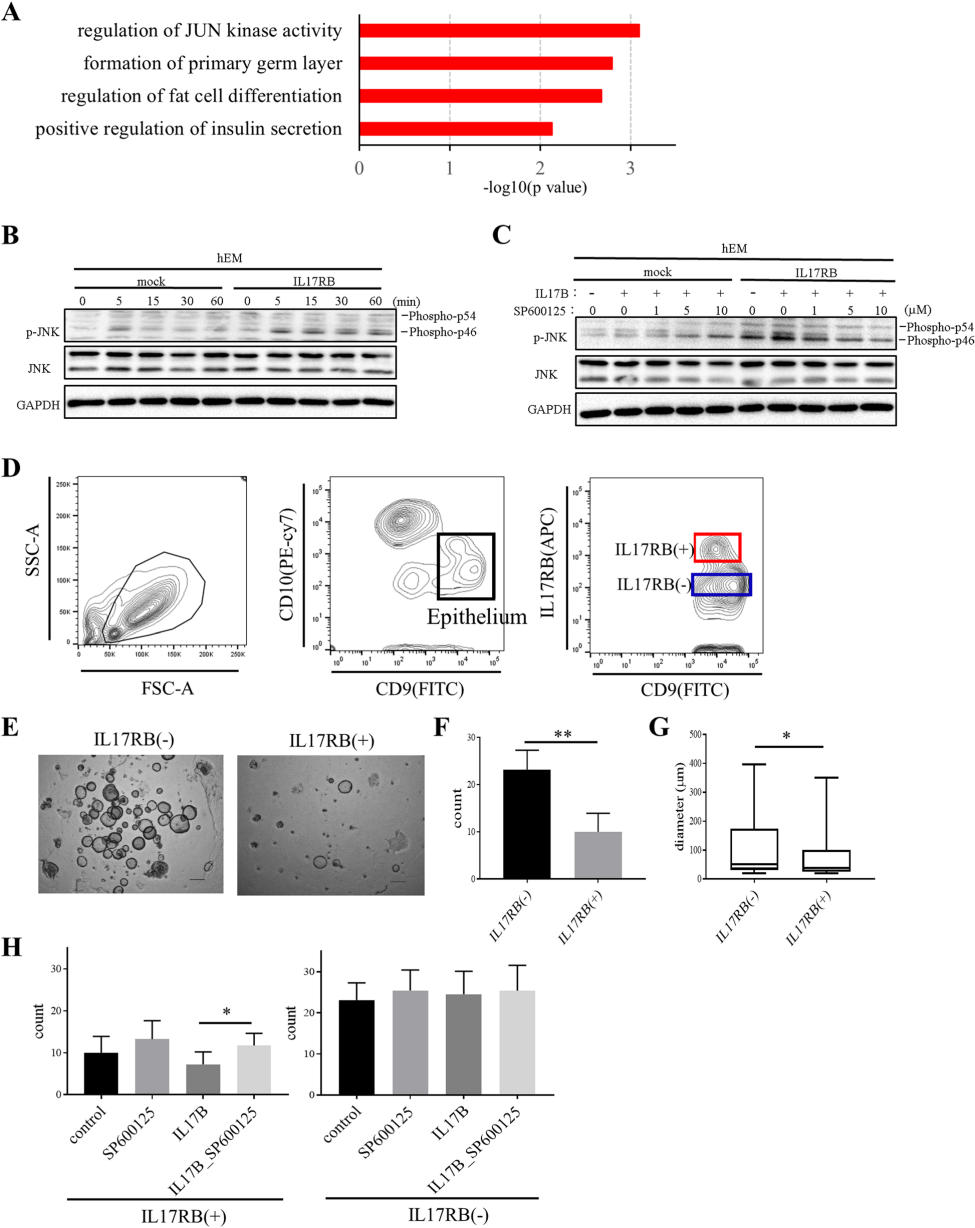
**A** Four Gene Ontology terms among 425 genes having significant associations (*P* < 0.001) with age-related differences in IL17RB expression in the murine uterus according to Pearson’s correlation analysis (data source: Kawamura et al. 2020). **B** Western blot time course (60 min) of JNK phosphorylation in IL17RB-hEM cells serum-starved for 16 h and then stimulated with IL17B (100 ng/ml). p-JNK: phosphorylated JNK; t-JNK: total JNK; GAPDH: loading control. **C** Western blot time course (5 min) of JNK phosphorylation in IL17RB-hEM cells serum-starved for 16 h and then stimulated with IL17B (100 ng/ml) plus SP600125 (0, 1, 5, 10 µM). p-JNK: phosphorylated JNK; t-JNK: total JNK; GAPDH: loading control. **D**–**H** Phenotypic differences associated with IL17RB expression in human endometrial epithelial cells. **D** Flow cytometry was used to sort and separate samples of endometrial cells (EM_9) by identifying a region with the classic signature of endometrial glandular cells (CD9^+^/CD10^−^) and subdividing it based on IL17RB expression. **E** Endometrial organoids on day 15 of culture. Scale bars: 300 μm. **F** Organoid count (diameter ≥ 20 μm) on day 15 of culture. Error bars denote standard deviation (*n* = 8 independent locations). ** *P* < 0.01. **G** Endometrial organoid diameter on day 15 of culture. * *P* < 0.05. **H** Organoid count (diameter ≥ 20 μm) in IL17RB(+) and IL17RB(−) cells cultured in ExM in the presence of SP600125 (5 µM), IL17B (100 ng/ml), or both. Error bars denote standard deviation (*n* = 8 independent locations). * *P* < 0.05

 Based on our findings, we hypothesized that signaling pathways associated with IL17RB expression are related to aging, and we tested this hypothesis using RNA sequencing data from our previous study (GEO accession number GSE132886): we compared total RNA in samples of uterine tissue from a group of aged mice (≥ 60 weeks old) with that in similar samples from a group of young mice (5 or 8 weeks old) [10]. We also tested all genes differentially expressed in these two groups for significant associations (*P* < 0.001) with differential IL17RB expression via Pearson’s correlation analysis. We subjected the 425 genes thus identified to ontology analysis [43], yielding four Gene Ontology (GO) terms with *P* value < 0.01(Supplementary Table SI). These results led us to focus on the JNK pathway as the most relevant to IL17RB (Fig. 5A).

The JNK pathway is a mitogen-activated protein kinase (MAPK) signaling pathway; JNK itself (a Jun amino terminal kinase) is a protein kinase that is activated in response to extracellular stress stimuli and inflammatory cytokines [44]. Functionally, it is involved in many cellular processes, such as proliferation, embryogenesis, and apoptosis. Our next step was to test whether the JNK pathway is upregulated by IL17RB expression in the human endometrium. We found that JNK phosphorylation (Phospho-p46) was enhanced by IL17B exposure (100 ng/ml), increasing over time in IL17RB-hEM more than in mock-hEM (Fig. 5B), but that this phenomenon was inhibited when SP600125, a JNK inhibitor, was also present (Fig. 5C).

### IL17RB-dependent traits in patient-derived EOs

We sorted the endometrial cells and separated them using flow cytometry to identify functional differences attributable to IL17RB expression. CD9 and CD10 are classic markers of endometrial epithelial and stromal identity, respectively [45, 46]. We thus considered cells in the CD9^+^CD10^−^ region to be endometrial epithelial cells and subdivided them based on IL17RB positivity (Fig. 5D). We embedded the resulting IL17RB(+) and IL17RB(−) subpopulations separately in Matrigel and cultured them in ExM, recording their growth for the first seven days via time-lapse photography (Video 1). On day 15 of the culture, we counted the number of organoids (diameter ≥ 20 μm) that had formed and measured their diameters. We observed significant reductions in organoid-forming capacity and proliferative potential in the IL17RB(+) cells (Fig. 5E–G), suggesting that IL17RB expression suppresses the ability of endometrial epithelial cells to form organoids and proliferate.

We also cultured these subpopulations separately in ExM in the presence of either a JNK inhibitor only (SP600125; 5 µM), IL17B only (100 ng/ml), both, or neither (control) while recording their growth via time-lapse video (Video 2–3). In terms of organoid count on day 15, organoid-forming capacity was slightly lower in IL17RB(+) cells exposed to only IL17B than in the unexposed condition (although this difference was not statistically significant). However, it was restored to levels comparable to the unexposed condition by the simultaneous addition of SP600125 + IL17B. In contrast, we did not observe any significant differences across exposure conditions in any of the IL17RB(−) experiments (Fig. 5H). These results suggest that IL17B suppresses organoid-forming capacity in IL17RB(+) cells via the JNK pathway.

### The JNK pathway is involved in endometrial senescence

**Fig. 6:**
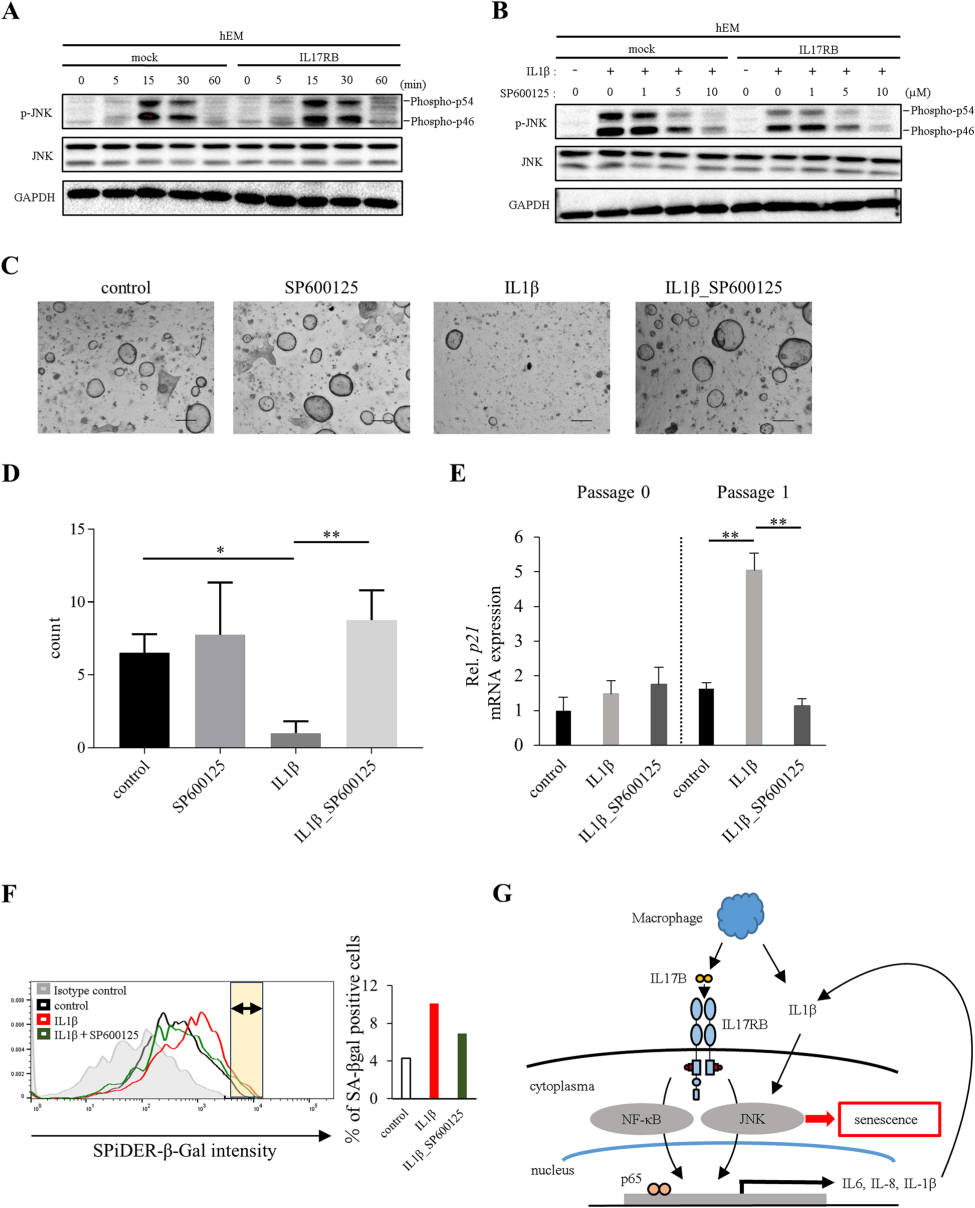
**A** Western blot time course (60 min) of JNK phosphorylation in IL17RB-hEM and mock cells serum-starved for 16 h and then stimulated with IL1β (100 ng/ml). p-JNK: phosphorylated JNK; t-JNK: total JNK; GAPDH: loading control. **B** Western blot time course (15 min) of JNK phosphorylation in IL17RB-hEM and mock cells serum-starved for 16 h and then stimulated with IL1β (100 ng/ml) plus SP600125 (0, 1, 5, 10 µM). p-JNK: phosphorylated JNK; t-JNK: total JNK; GAPDH: loading control. **c**–**e** Endometrial organoids (EM_10) cultured in ExM containing SP600125 (5 µM), IL1β (100 ng/ml), or both. **C** Passage 1 images. Scale bars: 300 μm. **D** Organoid count (diameter ≥ 20 μm; passage 1). Error bars denote standard deviation (*n* = 4 independent locations). **E** *p21* mRNA expression (passages 0, 1). Expression was quantified via RT-qPCR relative to an endogenous control (18 S rRNA). Error bars denote standard deviation (*n* = 3). * *P* < 0.05, ** *P* < 0.01. **F** SPiDER-β-Gal flow cytometry of endometrial organoids (EM_6, passage 2). Horizontal axis: SPiDER-β-Gal intensity; vertical axis: unit area. Bar graphs show the percentage of the area under each curve that is brighter than in the negative control (yellow area: SA-β-gal-positive cells). **G** Schematic of the endometrial senescence mechanism related to IL17RB

Next, we investigated the involvement of JNK signaling in the aforementioned mechanism of endometrial senescence caused by long-term IL1β exposure. We found that JNK phosphorylation (Phospho-p46, Phospho-p54) was enhanced by high IL1β exposure (100 ng/ml), increasing over time in both IL17RB- and mock-hEM treatments (Fig. 6A). These effects were inhibited when SP600125 was also present in the culture medium (Fig. 6B), indicating that IL1β does activate JNK in endometrial cells. We then cultured patient-derived EOs in ExM in the presence of SP600125 only (5 µM), IL1β only (100 ng/ml), both, or neither (control), and afterward evaluated their organoid-forming capacity. As in Fig. 2, organoid-forming capacity was significantly worsened by IL1β exposure in isolation (with respect to the control), but was rescued when SP600125 was added at the same time (Fig. 6C–D). Similarly, in passage 1, p21 expression was significantly upregulated in the IL1β-only condition, but this increase was suppressed in the IL1β + SP600125 condition, resulting in levels no different from the control group (Fig. 6E). Similarly, the proportion of SA-β-gal positive cells increased in the presence of IL1β (100 ng/ml), but the extent of the increase was diminished when IL1β was combined with a JNK inhibitor (SP600125, 5 µM) (Fig. 6F). Additionally, the rates of early and late apoptosis were no higher than in the control condition (Supplementary Fig. S7). These results suggest that IL1β-induced endometrial senescence is mediated by the JNK pathway.

Our data showed the endometrial senescence mechanism related to IL17RB. When endometrial cells expressing IL17RB are stimulated by IL17B, which is secreted by macrophages, they upregulate their expression of the SASP factors IL6, IL8, and IL1β. Of these cytokines, IL1β also induces endometrial senescence via the JNK pathway (Fig. 6G).

## Discussion

 Numerous studies have identified aging-related changes, which occur heterogeneously in different organ systems, as a risk factor for eventual organ dysfunction in a number of age-related diseases such as cardiovascular disease [47, 48], cancers [48–50], type 2 diabetes [51–53], and idiopathic interstitial pneumonia [54, 55]. In a previous study, we identified IL17RB as one of several markers of uterine aging based on its enhanced expression in the endometrial glandular cells of older women [10]. In the present study, we focused our attention on this cytokine receptor, analyzing its functional aspects in order to determine its effects on the endometrium. We observed NF-κB pathway activation and upregulation of several SASP factors (IL6, IL8, and IL1β) due to IL17B-specific binding in a human endometrial cell line forced to express IL17RB. The concept of “inflammaging” has gained currency in recent years, based on observations of increased prevalence and risk of inflammatory diseases with advancing age. Senescent cells induced by whole-organism stressors such as chronological aging, obesity, radiation, anticancer drugs, and oxidative stress are believed to provoke chronic inflammation, contributing to the pathological phenotype of various cancers and other age-related diseases. SASP factors induce senescence both in the cells themselves and in those that surround them via both autocrine and paracrine mechanisms, respectively [56, 57], as well as promoting chronic inflammation and carcinogenesis [56]. Few reports have analyzed the effects of SASP factors on the uterus, but some have found that the inflammatory response to bacterial infection of the uterus and vaginal lining can lead to endometritis and reduced receptivity [58, 59], suggesting that persistent bacterial exposure can induce the production of SASP factors and provoke chronic disease. Here, we used endometrial organoids as in vitro model for investigating how the endometrium responds to three specific cytokines whose expression is upregulated via IL17B/IL17RB signaling: IL6, IL8, and IL1β. We conducted experiments using endometrial tissues sourced from patients with leiomyoma or early cervical cancer. Leiomyomas are the most common benign gynecological tumor, affecting up to 80% of all women by the age of 50 [60], and many patients with leiomyoma carry pregnancies and give birth. Similarly, patients with early cervical cancer who have undergone trachelectomy to have their cervix removed have been reported to maintain a regular menstrual cycle and successfully conceive and deliver via cesarean Sect. [61]. These observations suggest that leiomyomas and early-stage cervical cancer cells have minimal impact on endometrial glandular function.

In the last decade there has been a dramatic increase in interest in using organoids derived from various tissues for aging research. Conventional two-dimensional (2D) cultures of established lines often use immortalized or tumor-derived cells, which are not suitable for research on aging. Organoids are considered more appropriate because they allow healthy, non-immortalized cells to be collected from people of different age groups and studied in experimental models where the in vivo environment and histological architecture can be more faithfully replicated [62]. Reductions in organoid-forming capacity with age have already been documented in murine organoids derived from intestinal epithelium [63] and pituitary stem cells [64]. Our study adds evidence that long-term IL1β exposure diminishes this ability in endometrial organoids, in addition to increasing β-galactosidase and p21 expression and inducing cellular senescence, all of which suggests that IL1β induces endometrial senescence via an IL17RB-related mechanism.

We also considered the source of the ligand of our receptor of interest, i.e., interleukin 17B. When dysregulated, lung commensal bacteria such as *Bacteroides* and *Prevotella* can provoke macrophages to produce IL17B via toll-like receptor (TLR)–Myd88 signaling, accelerating pneumonia and fibrosis as a result [65]. A similar mechanism may be at work in the endometrial environment, where bacterial flora similar to strains present in the alveoli have been identified [66]. We previously reported that endometrial macrophage populations increase significantly with age [10]; here, we have confirmed that IL17B is expressed inside endometrial macrophages and that circulating macrophages can be stimulated to produce it using a TLR4 agonist. It seems plausible that macrophages, after migrating to the endometrium in response to increasing aging-related inflammation, would secrete IL17B in their new environment, leading to endometrial aging.

The mechanism of macrophage IL1β secretion has been explained as follows: In response to various inflammatory stimuli, TLR signals are activated in macrophages, monocytes, and other inflammatory cells, inducing *IL1β* mRNA transcription, proIL1β production, and caspase-1 processing, thus yielding mature IL1β [67]. Our study confirms that macrophages derived from blood and endometrial tissue can produce this cytokine, suggesting that the macrophages that concentrate in the endometrium as part of normal aging exacerbate endometrial senescence by producing both IL17B and IL1β.

We sought to verify that IL17RB signaling induces senescence in endometrial cells by using the IL17RB-hEM model. Hypothesizing that signaling pathways associated with altered IL17RB expression are related to aging, we focused on the JNK pathway, which seemed to be the most relevant to IL17RB based on RNA sequencing data (GEO accession number GSE132886) from our previous study [10]. This pathway was activated in IL17RB-hEM in response to IL17B exposure, but this phenomenon was suppressed by the JNK inhibitor SP600125. IL17B also slightly reduced the organoid-forming capacity of IL17RB-positive cells isolated from endometrial tissue, and it was rescued by simultaneous exposure to SP600125. These findings indicate a connection between the JNK pathway and IL17B/IL17RB signaling, as well as the involvement of the former in organoid-forming capacity.

Several studies have already examined the JNK pathway in relation to aging. One found that hypoxia and reoxygenation activate it and accelerate synovial aging [68], while another noted that senescence phenomena in spermatogonial stem cells are caused by the activation of JNK-mediated glycolysis [69]. The JNK pathway is also apparently involved in the chemical reprogramming of somatic cells up to a certain point in the acquisition of stemness, and while pluripotent colonies proliferate after exposure to a JNK inhibitor, this behavior is suppressed by the addition of IL1β and TNF [70]. These findings position the JNK pathway as an important barrier to the reprogramming process. Our discovery that blocking JNK signaling in EOs acted to inhibit the signs of senescence induced by IL1β exposure provides further hints of this pathway’s role in endometrial senescence and regeneration.

## Conclusion

In the present study, we have revealed an association between IL17RB, whose expression increases in the endometrial glandular epithelium with advancing age, and cellular senescence. Using human endometrial organoids as in vitro model, we found that IL1β inhibits cell proliferation and leads to endometrial senescence via the JNK pathway. Further studies, including in vivo research, are needed to determine the role(s) played by the proposed mechanism of endometrial senescence in infertility, cancers, and other aging-related diseases of the uterus.

### Supplementary Information

Additional file 1: Fig. S1. (A) Schematic of organoid preparation. Endometrial tissue was harvested and treated with collagenase for 60–90 min to dissociate it into individual cells, which were then encased in Matrigel (seed density: 3.5 × 104 cells/culture). Organoids formed after 10–14 d, and medium was changed every 3 d. (b–c) Endometrial organoids and donor tissue (EM_1). Scale bars: 300 μm. (B) Image of organoids on day 14 of culture. (C) HE-stained sections of organoids and donor tissue (day 14).

Additional file 2: Fig. S2. (A–B) Endometrial organoids cultured in ExM containing IL1β. Bar graphs show organoid counts (diameter ≥ 20 µm) for the respective passages cultured in ExM with different concentrations of IL1β (0, 10, 100 ng/ml). Error bars denote standard deviation (*n* = 4 independent locations). Scale bars: 300 µm. (A) Patient: EM_3. Passage 0, 1, and 2 images were taken on day 18, 17, and 22 of culture, respectively. (B) Patient: EM_5. Passage 0, 1, and 2 images were taken on day 15, 13, and 12 of culture, respectively.

Additional file 3: Fig. S3. (A-B) SPiDER-β-Gal flow cytometry of endometrial organoids. Horizontal axis: SPiDER-β-Gal intensity; vertical axis: unit area. Bar graphs show the percentage of the area under each curve that was brighter than in the negative control (yellow area: SA-β-gal-positive cells). (A) EM_3, passage 2. (B) EM_4, passage 1. (C-D) p21 mRNA expression in endometrial organoids cultured in ExM containing IL1β (0, 10, 100 ng/ml). Expression was quantified via RT-qPCR relative to an endogenous control (18S rRNA). Error bars denote standard deviation (*n* = 3). (C) EM_2, passages 0, 3. (D) EM_5, passages 0, 3. * *P* < 0.05, ** *P* < 0.01.

Additional file 4: Fig. S4. (A) SPiDER-β-Gal flow cytometry of endometrial organoids (EM_6, passage 2). Horizontal axis: SPiDER-β-Gal intensity; vertical axis: unit area. Bar graphs show the percentage of the area under each curve that is brighter than in the negative control (yellow area: SA-β-gal-positive cells). (B) Apoptosis assay using endometrial organoids (EM_6, passage 2).

Additional file 5: Fig. S5. (A) Schematic of the differentiation protocol used to induce the production of macrophages from peripheral-blood monocytes. (B) Phase-contrast images showing the changes that occur over time during macrophage differentiation. Scale bars: 300 µm. (C) Flow cytometry histograms showing cell populations expressing two macrophage surface markers (day 7 of protocol).

Additional file 6: Fig. S6. (A) Bright field images of mock-hEM and IL17RB-hEM stained with SA-β-Gal. Top row: Passage 1, bottom row: Passage 30. Scale bar: 50 µm (B) Proportion of SA-β-Gal positive cells per unit area (2.25 x 10-12m2). **P 0.01. (C) p21 mRNA expression in mock-hEM and IL17RB-hEM cultured in DMEM/Ham's-F12 containing IL17B (0, 100 ng/ml). Expression was quantified via RT-qPCR relative to an endogenous control (HPRT1). Error bars denote standard deviation (*n* = 3). ** *P* < 0.01.

Additional file 7: Fig. S7. Apoptosis assay using endometrial organoids (EM_6, passage 2).

Additional file 8: Fig. S8. Original, uncropped immunoblots of Fig.1B, 1D, 5B-C and 6A-B. Red regions are saturated areas.

Additional file 9: Table. SI. Correlation analysis of all genes differentially expressed in two groups of aged and young mice for significant associations (*P* < 0.001) with differential IL17RB expression via Pearson's correlation analysis. We subjected the 425 genes thus identified to ontology analysis, yielding four Gene Ontology (GO) terms with *P* value < 0.01. 

Additional file 10: Video 1. The first seven days time-lapse videos showing organoid forming of IL17RB(+) and IL17RB(−) subpopulations.

Additional file 11: Video 2. The first seven days time-lapse videos showing organoid forming of IL17RB(+) subpopulations in the presence of either a JNK inhibitor only (SP600125; 5 µM), IL17B only (100 ng/ml), both, or neither (control).

Additional file 12: Video 3. The first seven days time-lapse videos showing organoid forming of IL17RB(-) subpopulations in the presence of either a JNK inhibitor only (SP600125; 5 µM), IL17B only (100 ng/ml), both, or neither (control).

## Data Availability

No datasets were generated or analysed during the current study.
