# Correction: Endometrial senescence is mediated by interleukin 17 receptor B signaling

**DOI:** 10.1186/s12964-024-01754-z

**Published:** 2024-07-22

**Authors:** Keiko Kawamura, Yumiko Matsumura, Teruhiko Kawamura, Hiromitsu Araki, Norio Hamada, Kazutaka Kuramoto, Hiroshi Yagi, Ichiro Onoyama, Kazuo Asanoma, Kiyoko Kato

**Affiliations:** 1 Department of Obstetrics and Gynecology, Graduate School of Medical Sciences, Kyushu University, Maidashi 3-1-1, Higashi-ku, Fukuoka, 812-8582 Japan; 2 Department of Business and Technology Management, Faculty of Economics, Kyushu University, Fukuoka, Japan

**Correction: Cell Commun Signal 22, 363 (2024)**.

10.1186/s12964-024-01740-5.

Following publication of the original article [1], the authors reported that the incorrect additional file 12 was published. The correct additional file is published in this correction article and the original article [1] has been corrected.

### Electronic supplementary material

Below is the link to the electronic supplementary material.

Additional file 12: Video 3. The first seven days time-lapse videos showing organoid forming of IL17RB(-) subpopulations in the presence of either a JNK inhibitor only (SP600125; 5 µM), IL17B only (100 ng/ml), both, or neither (control).
