# Supplementary figures and images for: Endometrial senescence is mediated by interleukin 17 receptor B signaling
Source: Cell Commun Signal. 2024 Jul 15;22:363. doi: 10.1186/s12964-024-01740-5 (PMC11247761; doi:10.1186/s12964-024-01740-5)

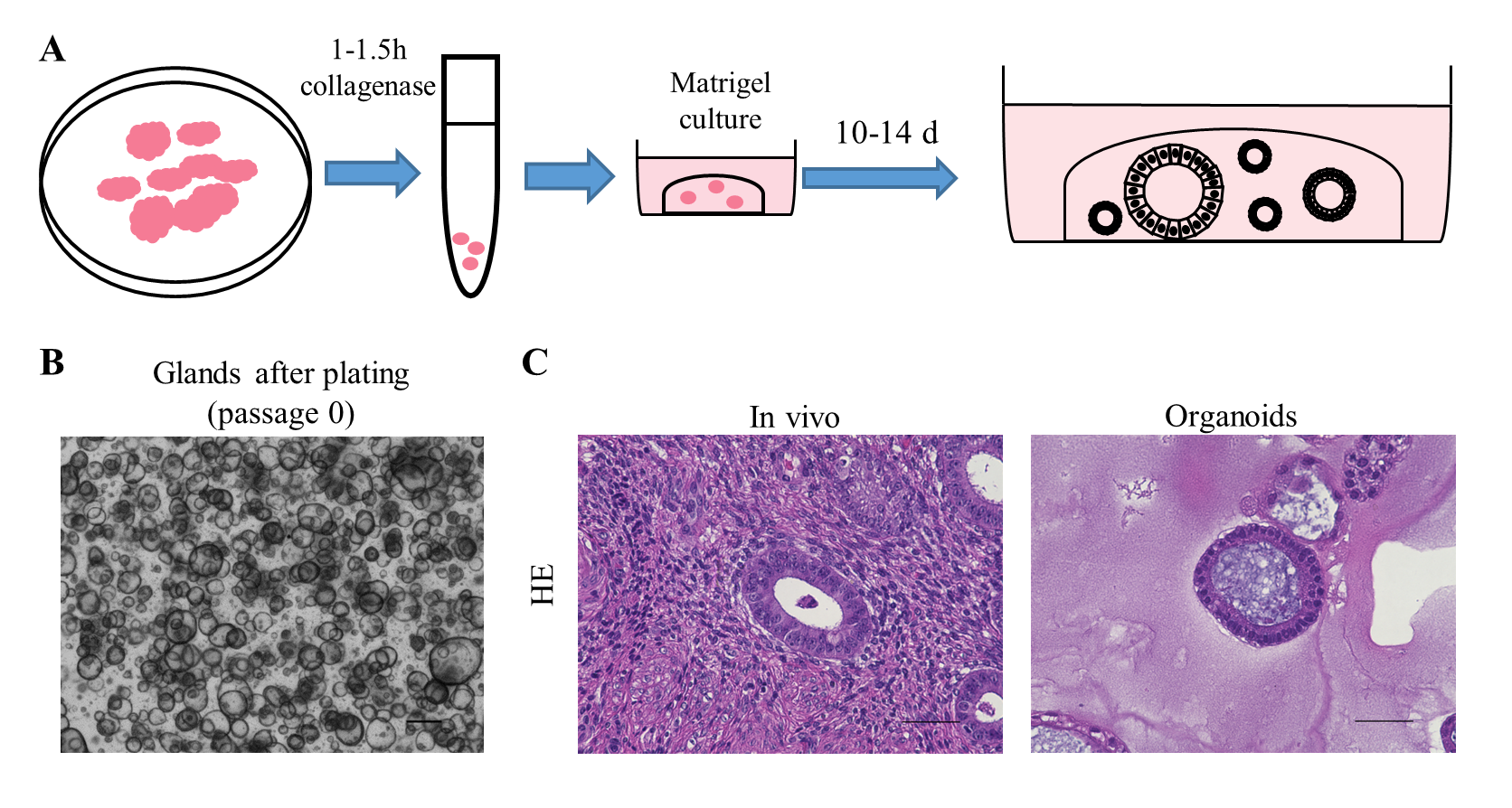

Supplement: Supplementary file 1 — Additional file 1: Fig. S1. (A) Schematic of organoid preparation. Endometrial tissue was harvested and treated with collagenase for 60–90 min to dissociate it into individual cells, which were then encased in Matrigel (seed density: 3.5 × 10^4^ cells/culture). Organoids formed after 10–14 d, and medium was changed every 3 d. (b–c) Endometrial organoids and donor tissue (EM_1). Scale bars: 300 μm. (B) Image of organoids on day 14 of culture. (C) HE-stained sections of organoids and donor tissue (day 14). [file 12964_2024_1740_MOESM1_ESM.tif]

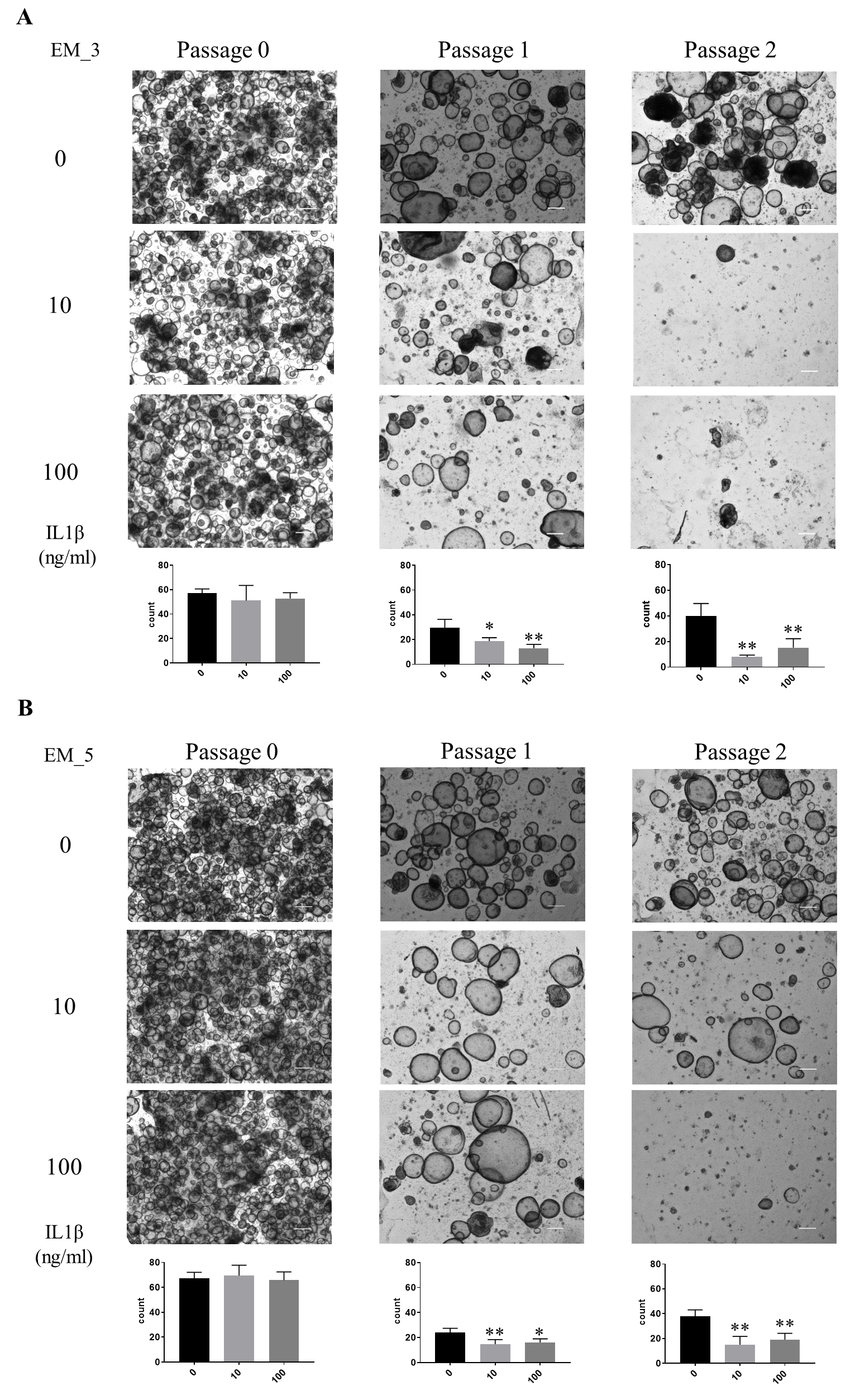

Supplement: Supplementary file 2 — Additional file 2: Fig. S2. (A–B) Endometrial organoids cultured in ExM containing IL1β. Bar graphs show organoid counts (diameter ≥ 20 µm) for the respective passages cultured in ExM with different concentrations of IL1β (0, 10, 100 ng/ml). Error bars denote standard deviation (n = 4 independent locations). Scale bars: 300 µm. (A) Patient: EM_3. Passage 0, 1, and 2 images were taken on day 18, 17, and 22 of culture, respectively. (B) Patient: EM_5. Passage 0, 1, and 2 images were taken on day 15, 13, and 12 of culture, respectively. [file 12964_2024_1740_MOESM2_ESM.tif]

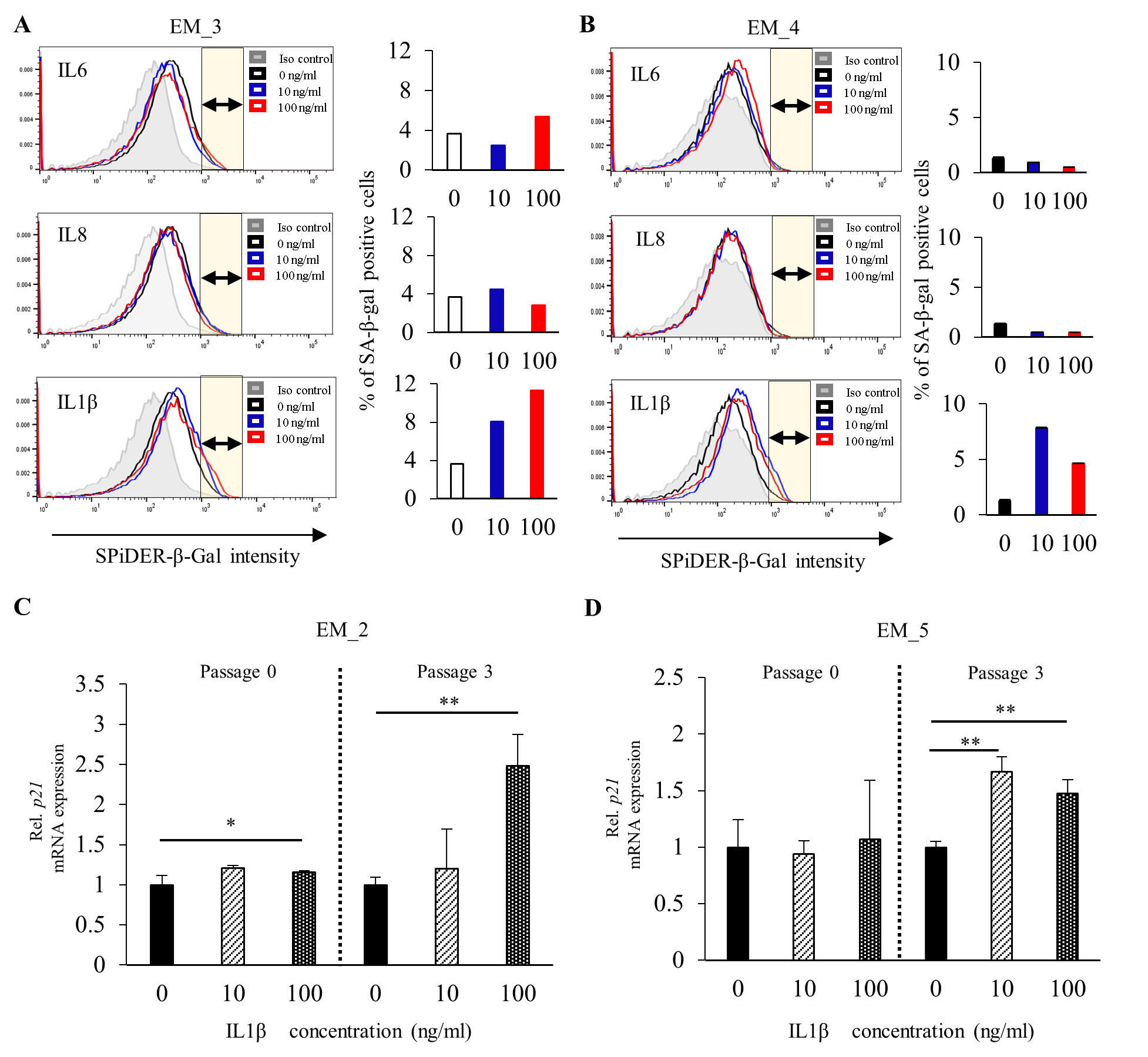

Supplement: Supplementary file 3 — Additional file 3: Fig. S3. (A-B) SPiDER-β-Gal flow cytometry of endometrial organoids. Horizontal axis: SPiDER-β-Gal intensity; vertical axis: unit area. Bar graphs show the percentage of the area under each curve that was brighter than in the negative control (yellow area: SA-β-gal-positive cells). (A) EM_3, passage 2. (B) EM_4, passage 1. (C-D) p21 mRNA expression in endometrial organoids cultured in ExM containing IL1β (0, 10, 100 ng/ml). Expression was quantified via RT-qPCR relative to an endogenous control (18S rRNA). Error bars denote standard deviation (n = 3). (C) EM_2, passages 0, 3. (D) EM_5, passages 0, 3. * P < 0.05, ** P < 0.01. [file 12964_2024_1740_MOESM3_ESM.tif]

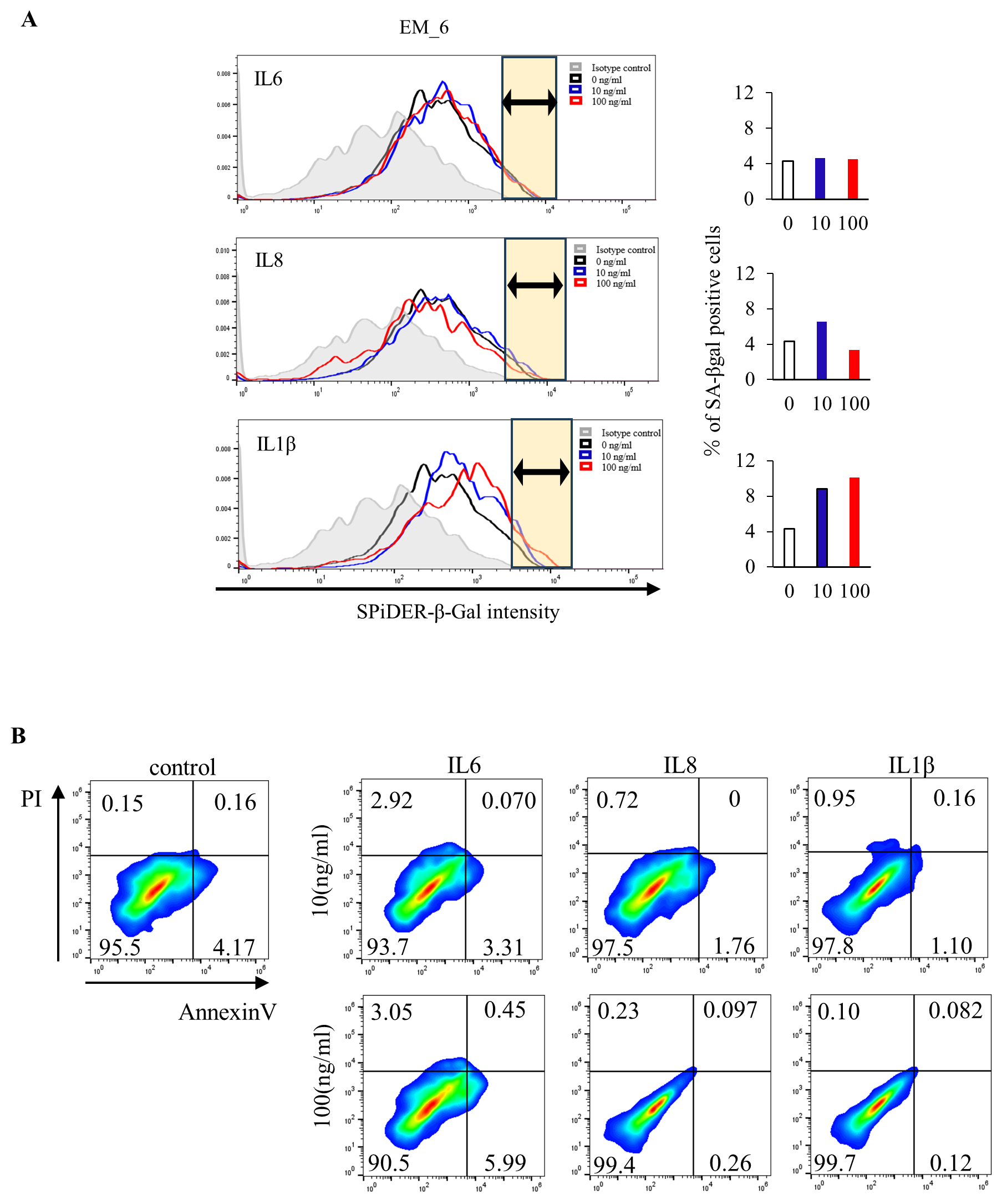

Supplement: Supplementary file 4 — Additional file 4: Fig. S4. (A) SPiDER-β-Gal flow cytometry of endometrial organoids (EM_6, passage 2). Horizontal axis: SPiDER-β-Gal intensity; vertical axis: unit area. Bar graphs show the percentage of the area under each curve that is brighter than in the negative control (yellow area: SA-β-gal-positive cells). (B) Apoptosis assay using endometrial organoids (EM_6, passage 2). [file 12964_2024_1740_MOESM4_ESM.tif]

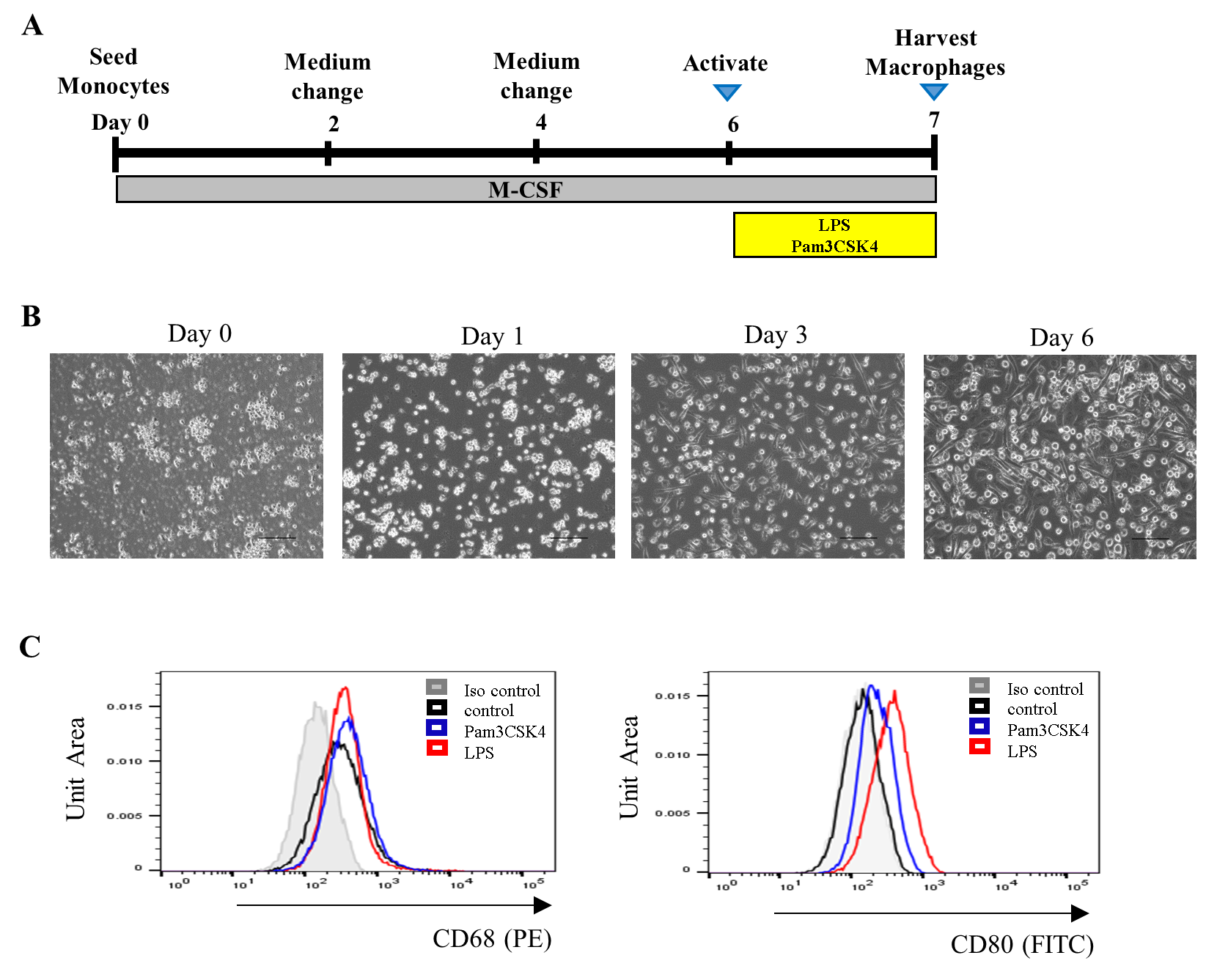

Supplement: Supplementary file 5 — Additional file 5: Fig. S5. (A) Schematic of the differentiation protocol used to induce the production of macrophages from peripheral-blood monocytes. (B) Phase-contrast images showing the changes that occur over time during macrophage differentiation. Scale bars: 300 µm. (C) Flow cytometry histograms showing cell populations expressing two macrophage surface markers (day 7 of protocol). [file 12964_2024_1740_MOESM5_ESM.tif]

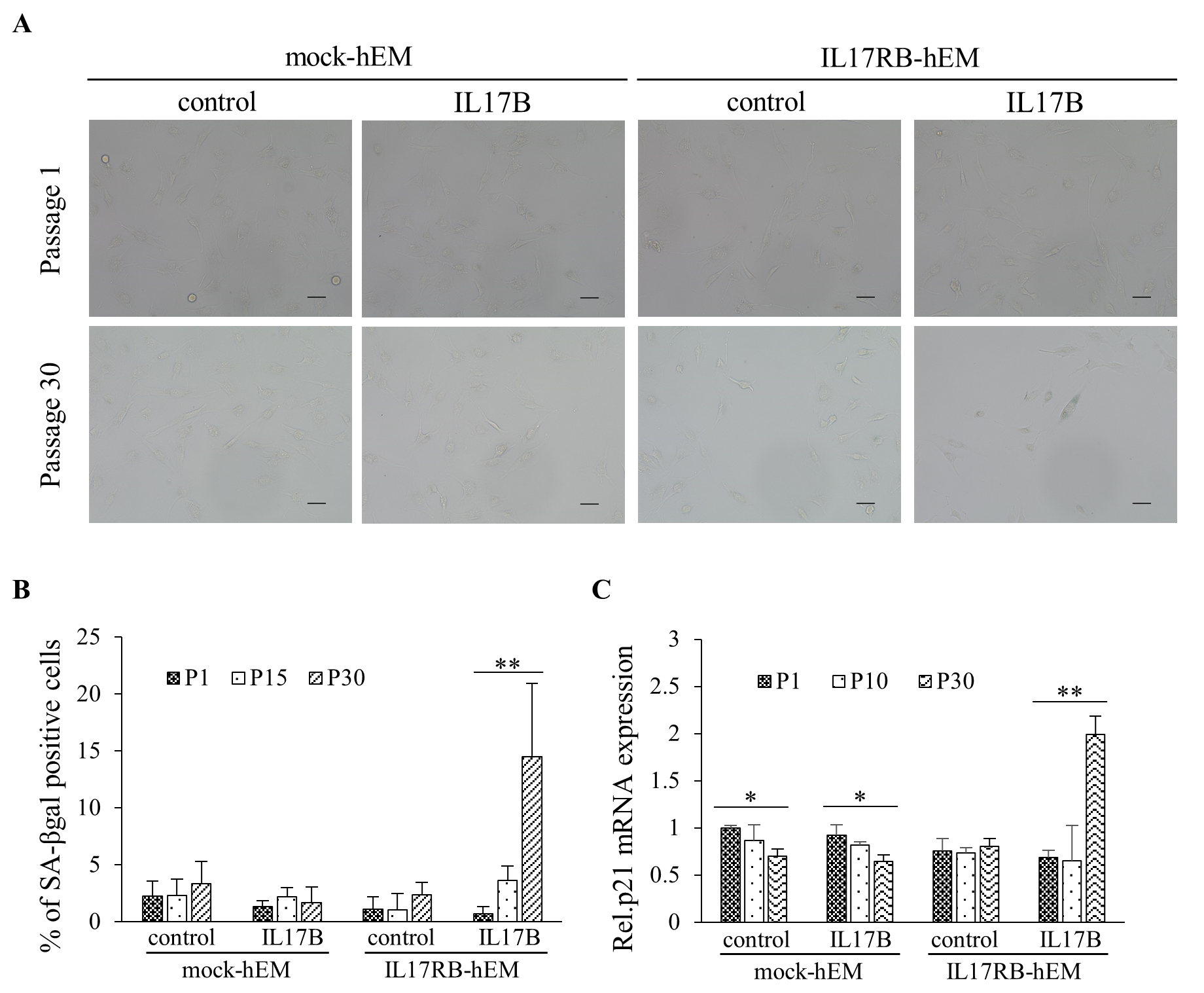

Supplement: Supplementary file 6 — Additional file 6: Fig. S6. (A) Bright field images of mock-hEM and IL17RB-hEM stained with SA-β-Gal. Top row: Passage 1, bottom row: Passage 30. Scale bar: 50 µm (B) Proportion of SA-β-Gal positive cells per unit area (2.25 x 10^-12^m2). **P 0.01. (C) p21 mRNA expression in mock-hEM and IL17RB-hEM cultured in DMEM/Ham's-F12 containing IL17B (0, 100 ng/ml). Expression was quantified via RT-qPCR relative to an endogenous control (HPRT1). Error bars denote standard deviation (n = 3). ** P < 0.01. [file 12964_2024_1740_MOESM6_ESM.tif]

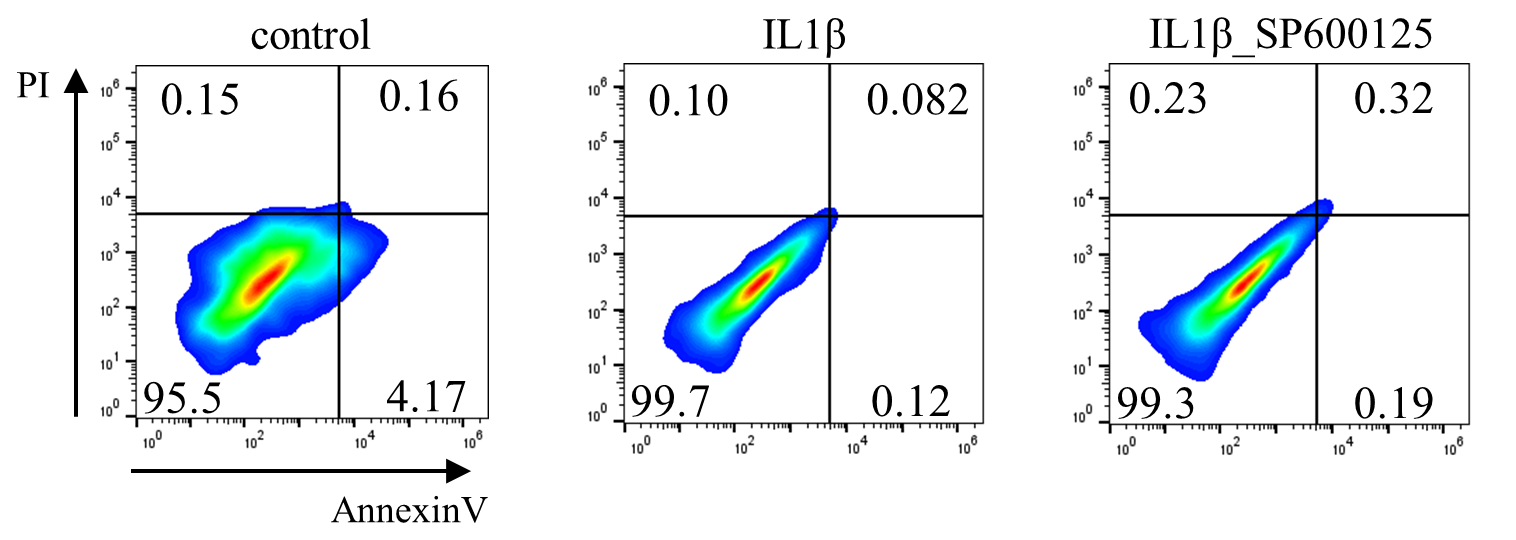

Supplement: Supplementary file 7 — Additional file 7: Fig. S7. Apoptosis assay using endometrial organoids (EM_6, passage 2). [file 12964_2024_1740_MOESM7_ESM.tif]

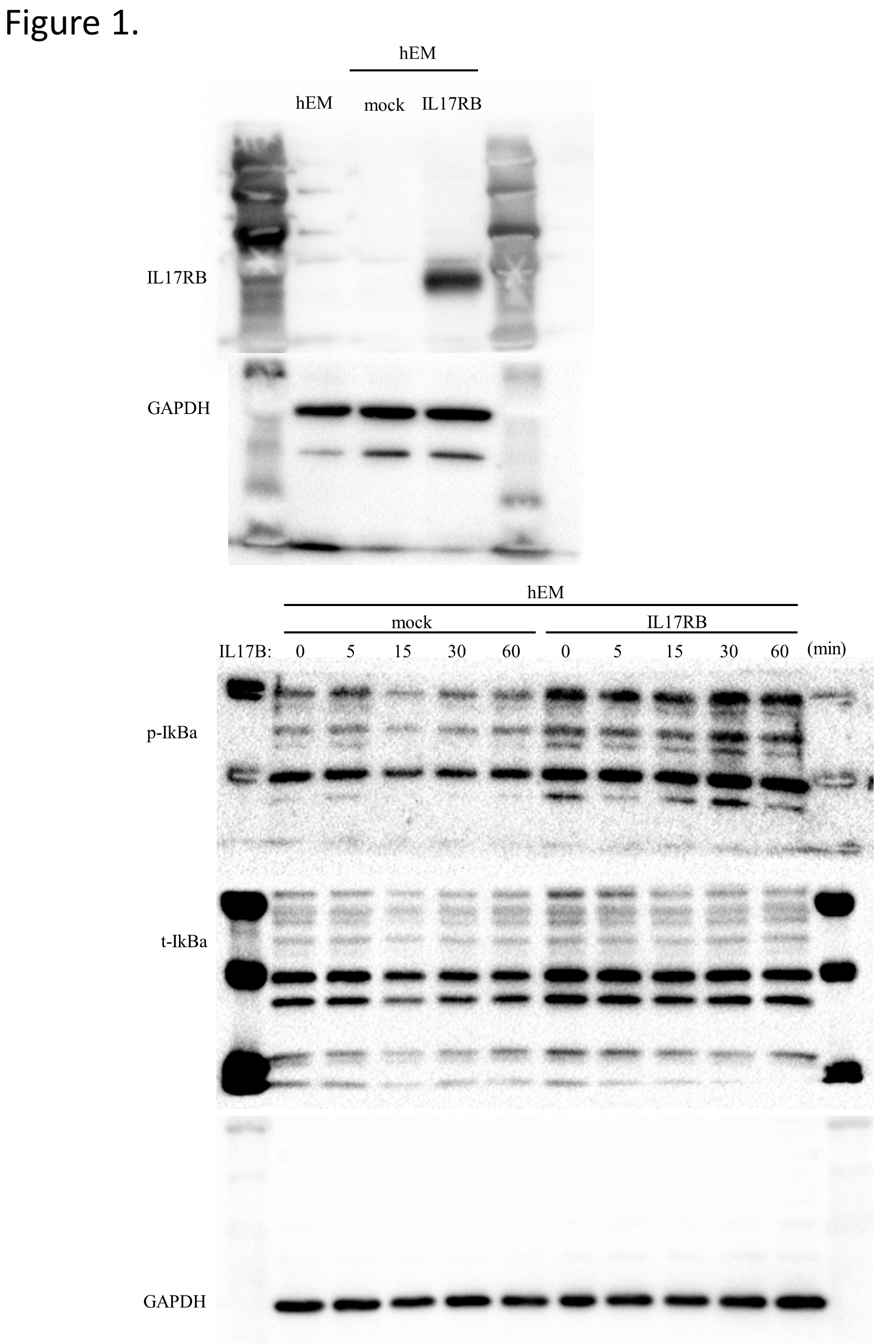

Supplement: Supplementary file 8 — Additional file 8: Fig. S8. Original, uncropped immunoblots of Fig.1B, 1D, 5B-C and 6A-B. Red regions are saturated areas. [file 12964_2024_1740_MOESM8_ESM.zip › Figure S8_1.TIF]

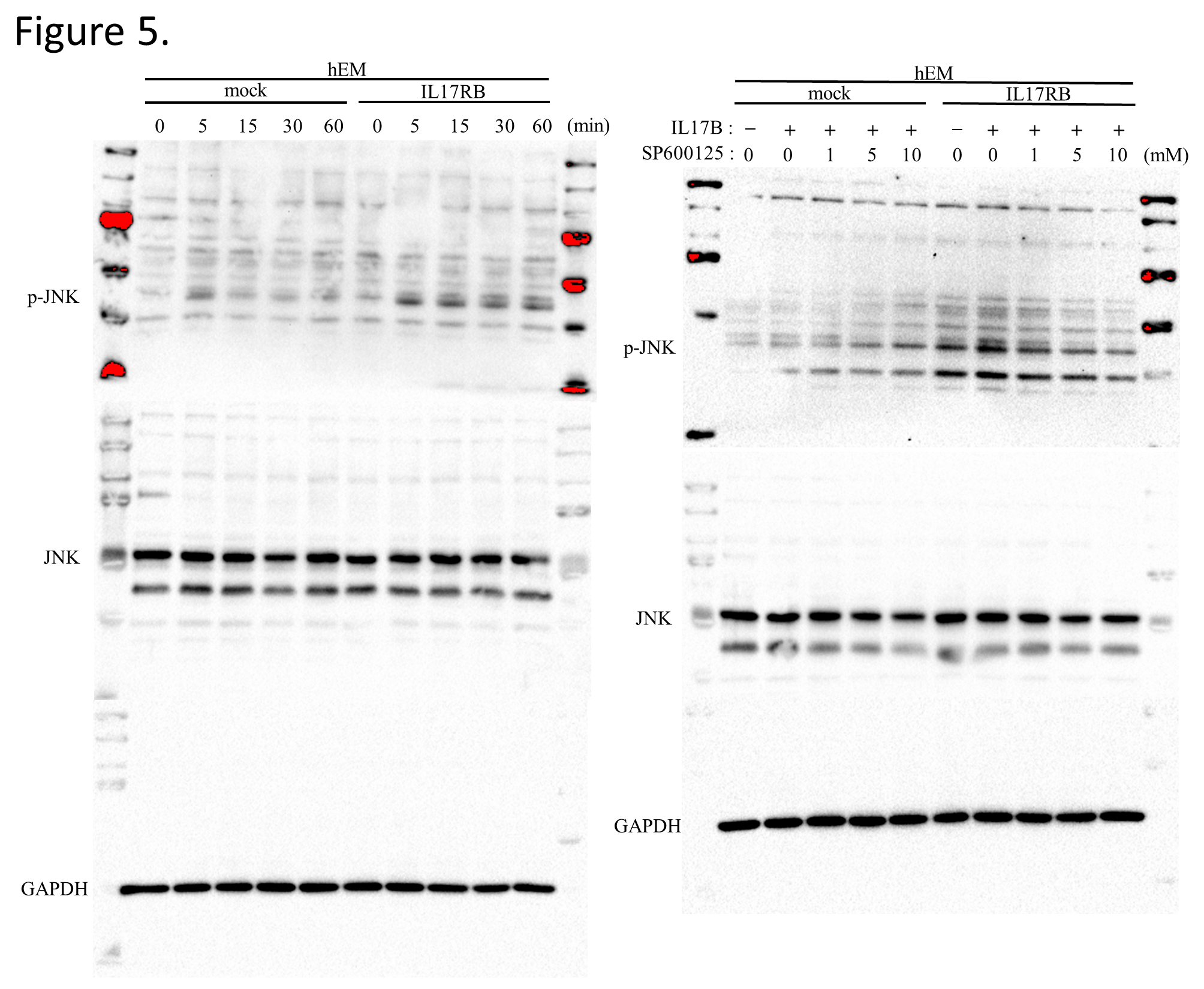

Supplement: Supplementary file 8 — Additional file 8: Fig. S8. Original, uncropped immunoblots of Fig.1B, 1D, 5B-C and 6A-B. Red regions are saturated areas. [file 12964_2024_1740_MOESM8_ESM.zip › Figure S8_2.TIF]

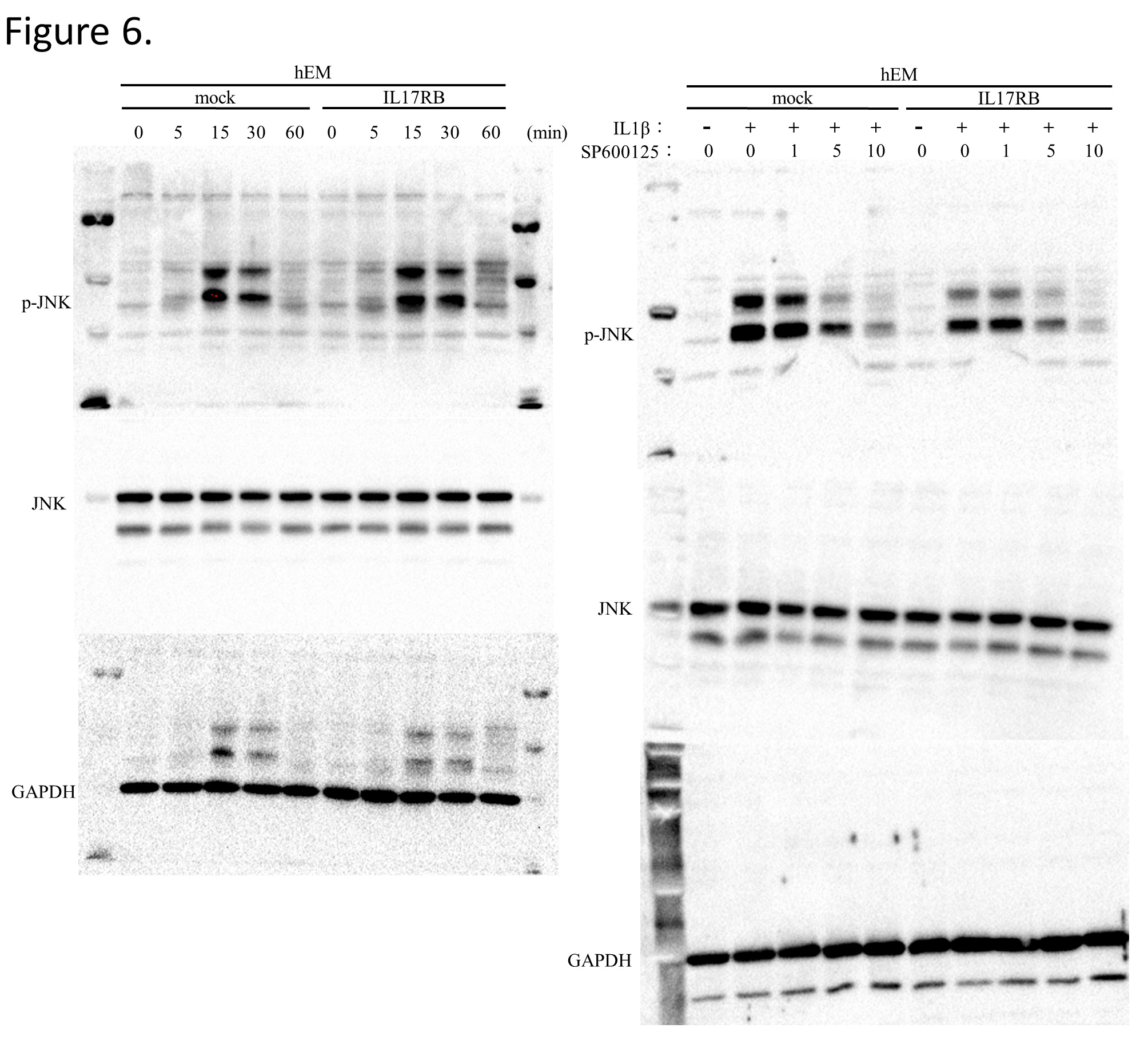

Supplement: Supplementary file 8 — Additional file 8: Fig. S8. Original, uncropped immunoblots of Fig.1B, 1D, 5B-C and 6A-B. Red regions are saturated areas. [file 12964_2024_1740_MOESM8_ESM.zip › Figure S8_3.TIF]
